# Focal Plant Observations as a Standardised Method for Pollinator Monitoring: Opportunities and Limitations for Mass Participation Citizen Science

**DOI:** 10.1371/journal.pone.0150794

**Published:** 2016-03-17

**Authors:** Helen E. Roy, Elizabeth Baxter, Aoine Saunders, Michael J. O. Pocock

**Affiliations:** 1 Centre for Ecology & Hydrology, Maclean Building, Benson Lane, Crowmarsh Gifford, Wallingford, Oxfordshire OX10 8BB, United Kingdom; 2 British Science Association, Wellcome Wolfson Building, 165 Queen's Gate, London SW7 5HD, United Kingdom; University of Northampton, UNITED KINGDOM

## Abstract

**Background:**

Recently there has been increasing focus on monitoring pollinating insects, due to concerns about their declines, and interest in the role of volunteers in monitoring pollinators, particularly bumblebees, via citizen science.

**Methodology / Principal Findings:**

The Big Bumblebee Discovery was a one-year citizen science project run by a partnership of EDF Energy, the British Science Association and the Centre for Ecology & Hydrology which sought to assess the influence of the landscape at multiple scales on the diversity and abundance of bumblebees. Timed counts of bumblebees (*Bombus* spp.; identified to six colour groups) visiting focal plants of lavender (*Lavendula* spp.) were carried out by about 13 000 primary school children (7–11 years old) from over 4000 schools across the UK. 3948 reports were received totalling 26 868 bumblebees. We found that while the wider landscape type had no significant effect on reported bumblebee abundance, the local proximity to flowers had a significant effect (fewer bumblebees where other flowers were reported to be >5m away from the focal plant). However, the rate of mis-identifcation, revealed by photographs uploaded by participants and a photo-based quiz, was high.

**Conclusions / Significance:**

Our citizen science results support recent research on the importance of local flocal resources on pollinator abundance. Timed counts of insects visiting a lure plant is potentially an effective approach for standardised pollinator monitoring, engaging a large number of participants with a simple protocol. However, the relatively high rate of mis-identifications (compared to reports from previous pollinator citizen science projects) highlights the importance of investing in resources to train volunteers. Also, to be a scientifically valid method for enquiry, citizen science data needs to be sufficiently high quality, so receiving supporting evidence (such as photographs) would allow this to be tested and for records to be verified.

## Introduction

Citizen science is defined as the involvement of volunteers in research [1, 2]. It has a long history [3, 4] but the past two decades have seen a rapid increase in the number of citizen-science initiatives [3–6]. Although citizen science initiatives span diverse areas of interest [1, 3, 7–9], biodiversity monitoring is particularly appealing for citizen science [1, 2, 4]. In the UK, the history of wildlife recording by expert volunteers is particularly rich [4, 10, 11]. Such expert volunteers have been making a major contribution to documentating change within the UK’s biodiversity and wider environment since the seventeenth century [11] and such volunteer activity has recently been considered as an example of citizen science [4]. In all cases citizen science can provide an effective means of combining ecological research with environmental education [12].

One of the reasons for the recent emphasis on citizen science is the recognition of its potential for supporting research in ecology: involving people who are not professional scientists permits the collection of greater volumes of data at larger scales and finer resolutions (both spatially and temporally) than would otherwise be possible or affordable [13, 14]. Other reasons include a growing confidence in the scientific accuracy and validity of public-generated datasets [15] and evidence of their use as valuable sources of information for addressing questions with scientific value [14, 16–21] and policy-relevance [22–25]. Technology available to support and inspire new citizen science initiatives is rapidly expanding [26, 27] and has enabled citizen science to become global in scale and engage hundreds of thousands of volunteers in many different scientific pursuits [10, 14].

Over the last ten years interest in the monitoring of pollinating insects has been growing [28, 29], due to concerns over their global declines [30] and impacts on their provision of ecosystem services [31]. Volunteer naturalists with high levels of taxonomic expertise have been compiling occurrence records of insect pollinators; such as in the UK through the activities of recording groups such as the Bees Wasps and Ants Recording Society http://www.bwars.com/ and the Hoverfly Recording Scheme http://www.hoverfly.org.uk/. The datasets collated through these volunteer networks have enabled the assessment of distribution change and trends for pollinating insects [30, 32]. However, there is an increasing emphasis on widening participation beyond the volunteer experts because this could support the collation of more data while engaging and educating many people.

Recently, pollinating insects have been the focus of several citizen science projects, due to a combination of the potential appeal to participants (i.e. the project will be successful in attracting participants) and the value of the data (i.e. the information collected is useful in the context of assessing pollinator declines) [33–36]. Projects have included: surveillance and monitoring of pollinating insects [13, 25, 37]; assessment of changes in nectar and pollen resources [38]; re-using data already collected to quantify trends [30]; understanding the ecology of the insects [39]; and assessing variation in pollination service [34, 40]. These citizen science projects have shown encouraging results both in engaging people with scientific concepts and deriving valuable scientific information. The specific methods vary but timed counts on focal plants [33, 34] represents a simple standardised method for monitoring flower-visiting insects, which are potential pollinators.

In this study, named the Big Bumblebee Discovery, we developed a method based on timed-counts of bumblebees (colour groups) visiting a focal plant (lavender, *Lavendula* spp.) as a standard floral resource. Our aim wasto engage a large number of primary school children (7–11 years old) to address a specific hypothesis: the diversity and abundance of bumblebees is influenced by the surrounding landscape at multiple scales—the large-scale (the wider landscape) and the fine-scale (the immediate surrounding floral resource). Our approach allowed us to address an hypothesis during a single field season while simultaneously testing this as a method for mass participation monitoring of pollinators. The Big Bumblebee Discovery enabled a timely critique of the opportunities and challenges inherent in mass participation hypothesis-led citizen science, as specifically applied to pollinator monitoring.

## Methods

### Project design

The Big Bumblebee Discovery was run by EDF Energy (a multinational company running this project as part of their school engagement programme) in partnership with the British Science Association (BSA: a science engagement charity) and the Centre for Ecology & Hydrology (CEH: a research institute). The BSA and EDF Energy invited primary schools across the UK, through their existing school networks, to participate in the Big Bumblebee Discovery and advertised the project widely to encourage participation from additional schools. Resources were provided through the EDF Energy portal called “The Pod”. They included a range of supporting materials such as citizen science lesson plans and teaching materials, identification posters, log books and fact sheets (S1–S4 Figs). Also, four podcasts (available to registered users of The Pod) introduced the project and its aims, and provided an overview of the methods. No further training was offered and no face-to-face training was provided. We did not gather information on the prior relevant knowledge of the participants or whether they had used the supporting resources. Registered schools received resources through the post including a standard (2-litre pot) lavender plant (*Lavandula angustifolia*), log books and identification posters (S1 Fig, S4 Fig).

Participants were asked to count bumblebees that visited a lavender plant over a measured time period (minimum of 5 minutes) during June to August 2014 and identify them according to six colour types. The reason that we use ‘colour types’ of bumblebees was to overcome the inherent difficulties of identifying the 24 species of bumblebee in Britain to species without microscopic examination. Identification of colour types is achievable in the field and provides a proxy for species diversity. Previous citizen science studies grouped bumblebee (*Bombus*) species into five distinct colour types [35, 39, 41] which we named: two-banded white tail, three-banded white tail, black-bodied orange tail, banded orange tail, and brown (Table 1), and we added a sixth type (orange white tail) because of the recent arrival of *Bombus hypnorum* to the UK [42].

**Table 1:** Distinction between species based on colour group based on [35, 39, 41].

Colour group	Abundant and widespread bumblebee (*Bombus*) species	Other bumblebee (*Bombus*) species, including cuckoo bumblebees (subgenus *Psithyrus*)
Two-banded white tail	*B*. *lucorum*, *B*. *terrestris*	*B*. *soroeensis*, *B*. *(P*.*) bohemicus*, *B*. *(P*.*) vestalis*, *B*. *(P*.*) sylvestris*, *B*. *(P*.*) campestris*, *B*. *(P*.*) barbutellus*
Three-banded white tail	*B*. *hortorum*	*B*. *ruderatus*, *B*. *jonellus*, *B*. *(P*.*) barbutellus*, *B*. *(P*.*) vestalisB*. *(P*.*) sylvestris*, *P*. *(P*.*) bohemicus*, male *B*. *lucorum*, *B*. *(P*.*) campestris*
Black-bodied orange tail	*B*. *lapidaries*	*B*. *ruderarius*, *B*. *(P*.*) rupestris*, *B*. *(P*.*) campestris*
Banded orange tail	*B*. *pratorum*	*B*. *monticola*, *B*. *sylvarum*, male *B*. lapidarius*, *B*. *ruderarius*, *B*. *(P*.*) rupestris*
Brown	*B*. *pascuorum*	*B*. *muscorum*, *B*. *humilis*, *B*. *distinguendus*, *B*. *(P*.*) campestris*
Orange white tail	*B*. *hypnorum*	

* Information in the artwork and accompanying notes provided to participants in this project clearly stated that a ‘banded orange tail’ has two yellow bands (on the abdomen and the thorax) while a bee with a single band on the front of the thorax (male *B*. *lapidarius*) should be recorded as ‘banded orange tail’, although we accept that misidentifications were possible.

Log books (S1 Fig) were provided to describe the required data fields, including: geographic location, description of location, date, duration of observations, weather, lavender type (English or French, i.e. cultivars of *Lavandula angustifolia* and *L*. *stoechas*, respectively), lavender patch size (width and depth in centimetres), position of lavender to nearest plants and bumblebee counts for each colour type. The large-scale landscape characteristics were quantified by the location description: urban, suburban, green space or countryside. The local-scale landscape characteristics were quantified as the distance to the nearest flowers: less than 30cm from other flowers; less than 1m from other flowers; less than 5m from other flowers; more than 5m from other flowers. Participants entered their results via a purpose-designed online form on ‘The Pod’ and were able to see real-time reporting of the results, standardised to counts per minute of observation, received from across the UK. Participants were invited to include photographs of the lavender plants and the bumblebees observed to allow verification of observations.

Each school registered via ‘The Pod’ was provided with a unique identification code for submitting records. Pupils were encouraged to take part during the weekends and school vacations and to use the unique identification code of their school when submitting records, although records with no unique identification code could also be submitted (e.g. from those without a school registration or from those who had forgotten their code). The fact that identification codes were at the level of the school rather than for individual teachers or children meant that we could not directly calculate the number of participants, although this could be estimated because the number of observers was entered with each record.

### Verification of records

We verified the identification of the photographs uploaded by participants, which allowed us to assess the accuracy of this subset of records. We reported accuracy as precision (the proportion of records submitted as such which were correctly identified) and miss rate (the proportion of records of that type which were not correctly identified, where miss rate = 1 –sensitivity). Comparison of the number of colour types reported against the number of individual bumblebees per report provided some additional indication of the confidence we could have in the results.

We also undertook an independent assessment of the ability of untrained observers to identify bumblebee colour types through a quiz for participants within a workshop at the British Science Festival (Birmingham, September 2014). The quiz included photographs of 30 insects each shown for 8 seconds. Participants recorded their identification based on reference to the guides produced for the Big Bumblebee Discovery. The quiz included different numbers of photos of different colour types (Table 2) plus *Bombus vestalis* (1 photo), a social wasp (*Vespula* sp.; 1 photo), honey bee (*A*. *mellifera*; 2 photos), *Eristalis* hoverfly (2 photos) and a butterfly (1 photos). The butterfly was included as the first non-bumblebee photo so as to indicate to participants that not every photo would be a bumblebee. 27 people participated in the quiz, including both adults and children. They were provided with the Big Bumblebee Discovery identification sheets (S2 Fig) but no additional training, in order to mimic the conditions of Big Bumblebee Discovery participants in the field.

**Table 2:** Classification of the verifications based on photos that were uploaded by participants and on identification of photographs by seminar attendees at the British Science Festival. Precision is the proportion of submitted identifications which were correct; miss rate is the proportion of records which were of a colour group but were incorrectly identified. Precision was separated as into ‘colour type’ and bumblebee, i.e. the proportion of records submitted as that colour type which were correct to colour type or correctly identified as a bumblebee, respectively. The full mis/classification matrix is in S2 Table.

True colour type	Photos submitted by participants in the Big Bumblebee Discovery (N = number of photos received identified as such)				Results of quiz undertaken by 27 attendees (adults and children) at a seminar at the British Science Festival, aggregated across all participants (N = number of photos in the quiz)			
	N	Precision (colour type)	Precision (bumble-bee)	Miss rate	N	Precision (colour type)	Precision (bumble-bee)	Miss rate
Banded orange tail	14	0.31	0.50	0.17	5	0.61	0.96	0.36
Black-bodied orange tail*	7	0.86	0.86	0.40	3	0.80	0.96	0.29
Brown	5	0.0	0.60	1.00	1	0.23	0.30	0.36
Orange white-tail	24	0.08	0.75	0.50	2	0.56	0.83	0.39
Two-banded white tail	24	0.81	0.92	0.22	6	0.72	0.99	0.36
Three-banded white tail	0	NA	NA	1.00	4	0.53	0.99	0.53
Unknown bbee	5							

* For submitted photos of black-bodied orange tails (*Bombus lapidarius*), the miss rate of identification of females was 0.14, whereas for males it was one. For the quiz photos, the miss rate of identification of females was 0.06 (two photos presented), whereas for males it was one (one photo presented).

### Analysis

This project was explicitly presented to participants as a project to collectively gather data and test hypotheses: that the richness and abundance of bumblebee colour types was influenced by the landscape surrounding the focal lavender plant at multiple scales (the large-scale, i.e. landscape type, and the fine-scale, i.e. distance to nearby flowers). However, due to our lack of confidence in the accuracy of participants’ identifications (see Results) we restricted ourselves here to assessing the impact of landscape context on reported overall abundance of submitted records, i.e. reported bumblebees.

Reports of bumblebees were from varying sized patches of lavender and over varying lengths of time. Therefore, counts were converted to a standard rate of counts of all reported bumblebees in 5 minutes in the average-sized lavender patch (37cm diameter: average obtained from records submitted to the Big Bumblebee Discovery). We removed two records with incorrect time data and eight records where the width and depth were both recorded as 1cm.

We used a generalised linear mixed model in R 3.1.2 to assess the impact of the factors of large-scale landscape (4 levels) and distance to the nearest plants (4 levels) on the log-transformed rate of total bumblebees reported, adding a small number (0.01) to avoid rates of zero. Location (100m grid reference) was used as a random effect, to take account of the pseudoreplication of multiple observations per school per day. Date was included as a quadratic relationship to permit a peak in the totals). The landscape characteristics at each scale were included as factors (with four levels each) rather than covariates because we wanted to include non-linear effects and because the wider landscape descriptions were not inherently ordered. There were too few combinations between all the factors to robustly include their interactions in the model.

### Community-led hypothesis testing

The results were presented in a workshop at the British Science Festival (September 2014) and we invited the workshop participants to collectively suggest additional hypotheses to explore using the Big Bumblebee Discovery dataset. From the suggestions we chose to test the effect of the type of lavender on the recorded bumblebee abundance and included this in all analyses. While this does not represent truly co-created citizen science [12], it did involve a small sample of non-expert volunteers more deeply in consideration of the data, by proposing hypotheses *post hoc* to data collection.

## Results

### Participation

**Fig 1:**
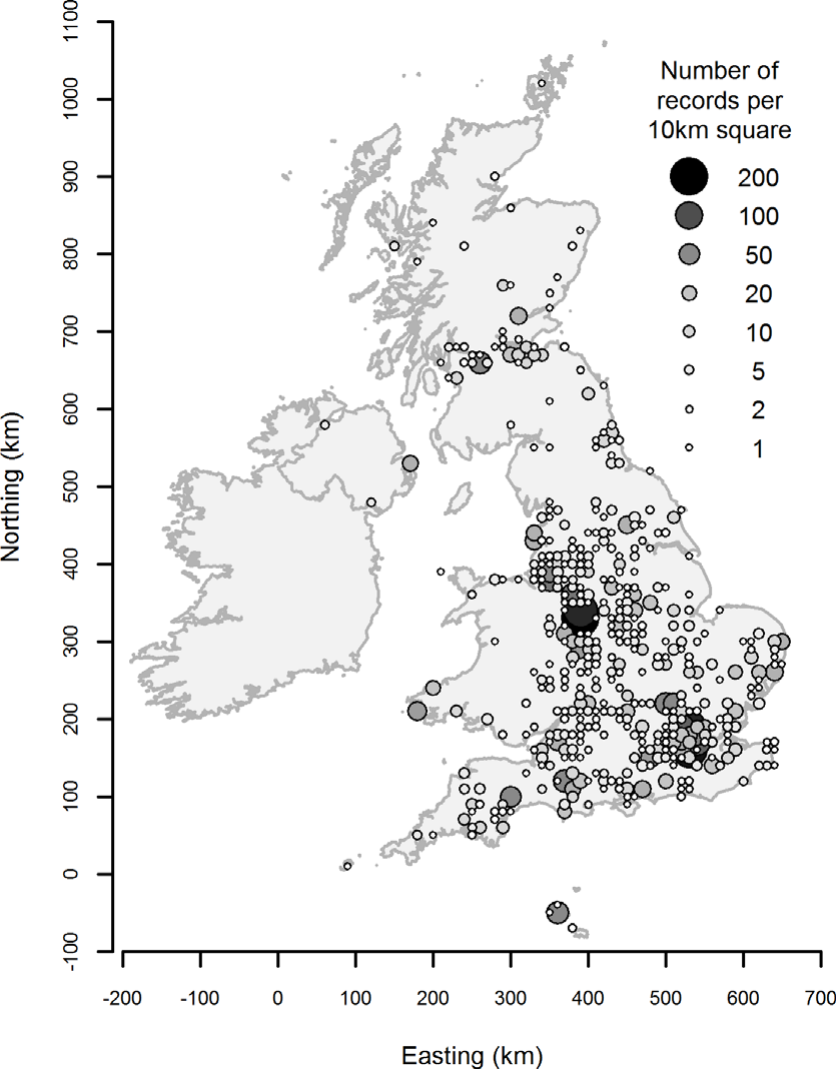
The number of records received per 10km grid square in the UK to show the spatial spread of participation.

A total of 3948 reports were received constituting a total of 26868 bumblebees, of which 3556 records were valid for analysis (i.e. no missing data or extreme outliers). Records came from 1561 unique locations (where each location is a 100m grid square) scattered across the UK (Fig 1); S1 Table).

We estimate that 13 024 children participated in the project, from at least 400 schools. Specifically, records were submitted with 400 unique identification codes, plus 768 records without a unique identification code; so our estimate assumes an average of 30 children took part per school and each record without a unique identification code was from a different group of observers. The maximum and minimum potential number of participants was 30 077 and 4646 (respectively assuming each record was from a unique group of observers or each record sharing an identification code was from the same group of observers).

Most people reported doing the project in pairs (22% of submissions), on their own (18%) or in groups of 3 or 6 (9% each). An approximately equal number of records was received from participants using pots of lavender supplied by EDF Energy (39%) and a patch of lavender planted in the ground (42%), while a smaller number were from the participant’s own pots of lavender (19%). Records were received from across the UK, but the density of records per location varied considerably (due to a combination of the number of groups of observers per school and the number of times the surveys were repeated). There tended to be fewest reports in areas of low human density, as we expected.

### Accuracy of the data

Only a small number of photographs (83) were uploaded with the submissions of records. The precision of these submissions varied according to the colour type being lowest for ‘brown’ bumblebees (none of the 5 identified correctly) and highest for ‘black-bodied orange tail’ and ‘two-banded white tail’ (81–86% identified correctly) (Table 2; full tables in S2 Table). Common bumblebee species in these colour groups (*B*. *lapidarius* and *B*. *terrestris/lucorum*, respectively) are large, distinctive species and so this results is not surprising, whereas species in the other colour groups fit less well to the expected stereotype of bumblebees. The proportion of these photographs correctly identified as bumblebees was high (precision = 75%) although there were still substantial misidentifications (e.g. half of the photographs identified as ‘banded orange tail’ were not bumblebees). Verification of the photographs showed that these photos included several other types of insect, particularly honeybees (*Apis mellifera*; 6% of photos with an identifiable insect) and large hoverflies (e.g. *Eristalus* spp.; 8%) (S2 Table). Generally, lavender was correctly identified (high precision); both photos of French lavender were correctly identified and 84% of the 51 photos submitted as English lavender were correct. The miss rate for bumblebees (the proportion of photos of a colour group which were correctly identified as such) varied substantially across colour groups from low (banded orange tail and two-banded white tail) to very high (brown and three-banded white tail).

These results were matched by those from the quiz at the British Science Festival workshop; namely precision (the proportion of submissions which were correctly identified) varied across colour groups, being lowest for the ‘brown’ colour type and highest for ‘black bodied red tail’ and ‘two striped white tail’ (Table 2). However, miss rate varied less strongly across the colour groups with 29–53% of the photos of each colour group being incorrectly identified.

The overall precision of identification to the colour group was 44% for field observations and 57% for the photo quiz. However, precision varied substantially across the colour groups.

**Fig 2:**
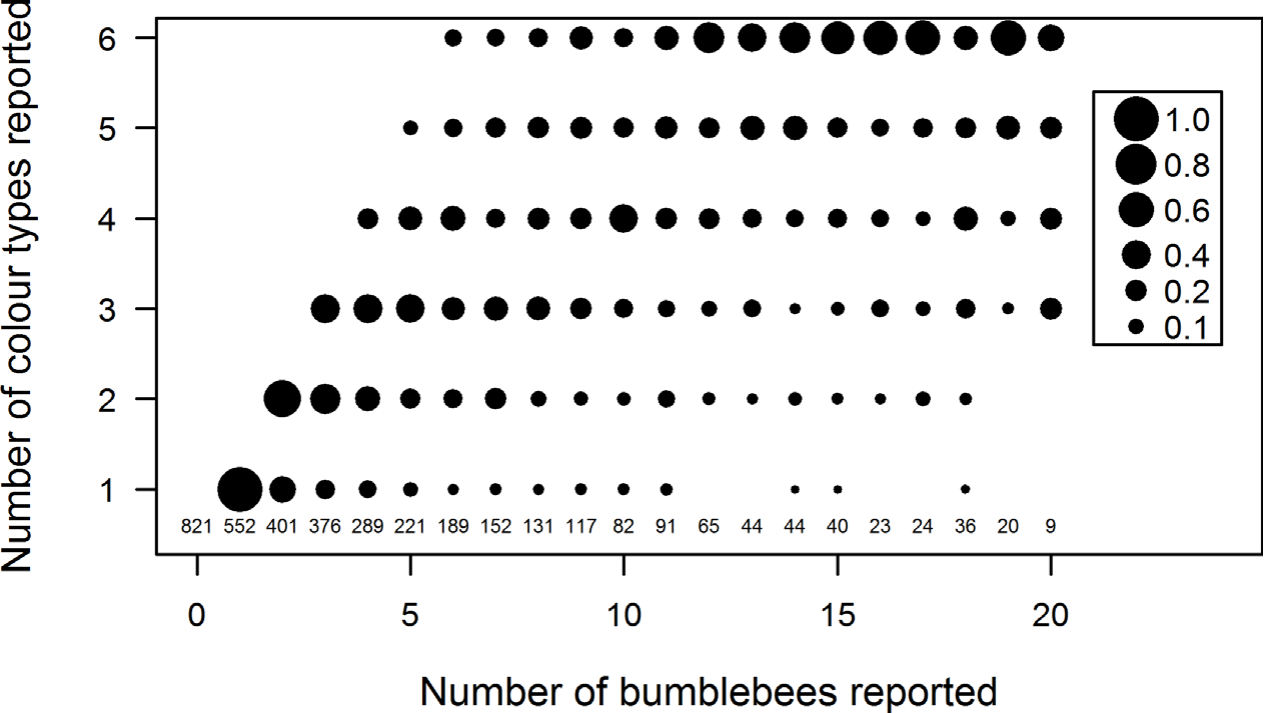
The proportion of the number of colour types reported according to the number of bumblebees counted per observation. Only observations up to counts of 20 are shown. The rapid rise in the number of colour types reported with increasing number of observations, and the large proportion of observations with all six colour types reported, suggested there was a propensity to over-report the true number of colour types, although this could not be independently verified.

We expected that the richness of bumblebee colour type per report would be much less than six. However, the results showed that a large proportion of counts were of 5 or 6 colour types and relatively few counts of more than five bees were of just one or two colour types (Fig 2). This observation provides circumstantial evidence that children were more likely to record multiple colour types than we expected. Our observation of school children’s participation showed that while the children were careful observers, some sought to hunt for all the colour types, a situation that was likely to lead to over-reporting of the richness of colour types.

### Rate of observations and weather

**Fig 3:**
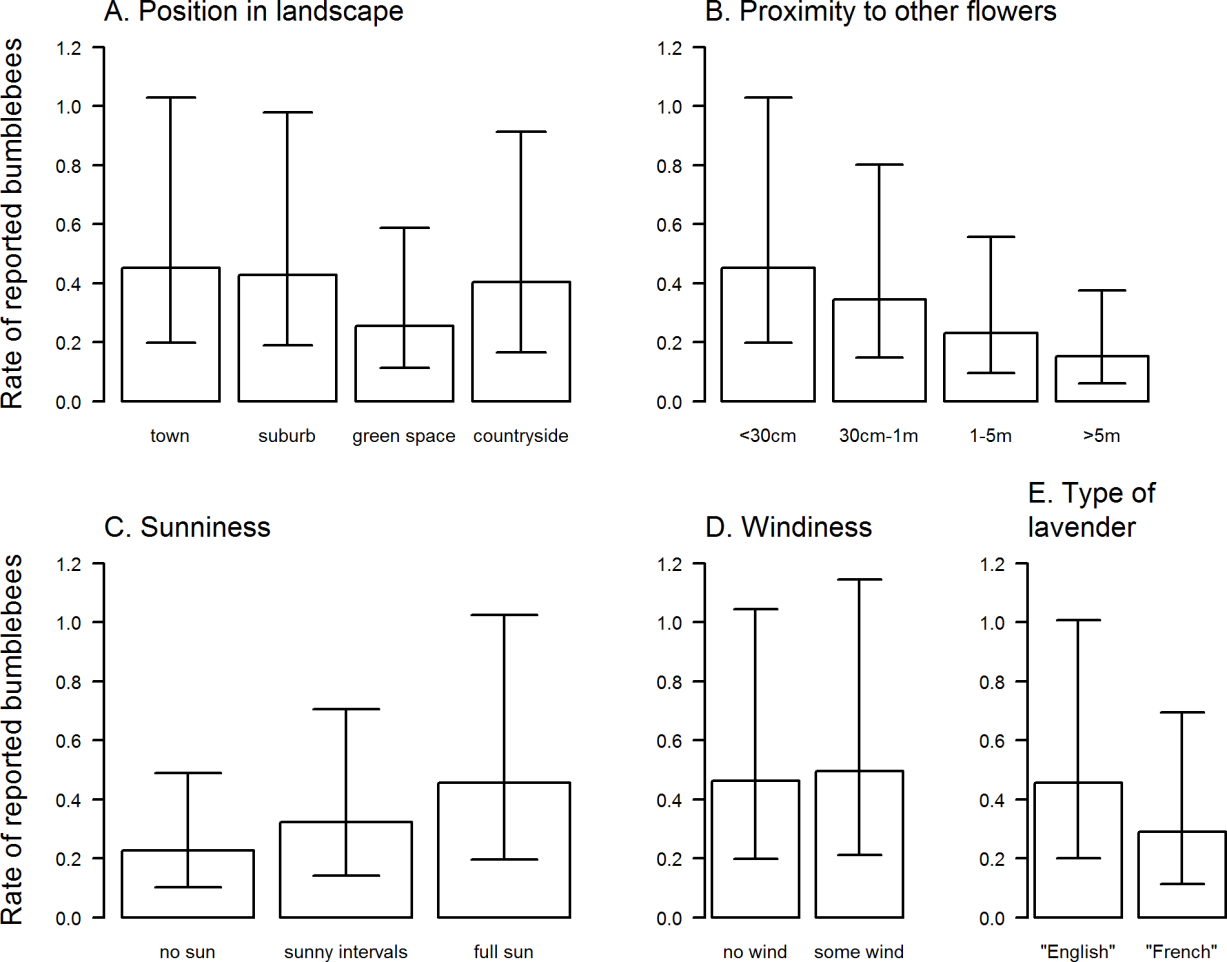
The importance of the different variables on the reported rate of bumblebees (standardised for five minutes on a lavender 37 cm in diameter, i.e. the average in this study). Except for variation in the variable of interest, the rates are shown relative to locations in towns, very close (<30cm) to other flowers, on English lavender and on days with full sun and no wind.

Due to our lack of confidence in the accuracy of records to colour type, we undertook analysis only of the totals of individuals reported, rather than the number of colour types reported. In doing this we assumed that the rates of misidentification of bumblebees were randomly distributed across the sample. For observations from towns, close to other flowers (<30cm), on days with full sun, the average rate of reported bumblebees was 0.4 per 5 minutes at an average-sized plot of lavender (37cm diameter). The analysis showed that the wider landscape type had no significant effect on reported bumblebee abundance (P = 0.157; Fig 3A), although point estimates of abundance indicated that towns and suburbs had, on average, higher rates of visitation than green spaces in towns or the countryside, which broadly fitted our expectations. The proximity to flowers was significant (P < 0.001; Fig 3B), with an almost halving of the rate for lavender >5m away from other flowers compared with lavender next to other flowers (Fig 3B). Increased sunniness had a highly significant effect on the rate of bumblebees reported (P<0.001; Fig 3C); with an almost doubling for counts taking place in full sun compared to counts taking place during no sun. There was no significant effect of wind (P = 0.131; Fig 3D) or species of lavender (P = 0.945; Fig 3E).

## Discussion

The Big Bumblebee Discovery is an example of a citizen science project which engaged a large number of participants from schools (estimated 13 000 school pupils aged 7–11 years old) in gathering data in a standardised way with timed, focal plant observations of pollinators in order to answer a specific hypothesis. The results from our analysis that were significant were not surprising: counts of reported bumblebees were highest on sunny days and when the focal plant was near other flowers. However, this study was the first to empirically test the accuracy with which members of the public can identify bumblebee colour types. We found that precision in identifying insects as bumblebees was 75% (i.e. one in four reports of a bumblebee was not a bumblebee) but that precision in identifying colour types was much lower (down to 43% for field observations and 57% for the photo quiz). Our study shows that care needs to be taken when engaging untrained participants that lack experience, even when providing relatively simple identification tasks, in order to ensure that the data are fit for the intended purpose.

It was surprising to discover the problems experienced by participants in accurately identifying colour types of bumblebees because such coarse-level identification is not often considered to be a major limitation in citizen science. One, widely-cited study provides the positive conclusion that “citizen scientists with modest training can collect useful observational data [on pollinators]…” [43] although, to put that study in context, most of the 13 participants had undergraduate degrees and each received 2 hours of face-to-face training. Previously, identification of bumblebees to colour type have been assumed to be correct [39]; although in that case the observers were householders observing bumblebee nests. The participants and context of each study will undoubtedly have an effect on rates of accuracy, but accuracy must be assessed. Our results were more in line with another citizen science study of pollinators at focal plants in which 34% of insects were mis-identified [37], although the authors do not describe the types and degree of error.

The inaccuracies in our study appeared to be due to the difficulty of identifying distinct colour groups (as supported by the quiz results) possibly compounded with a tendency to over-record richness. It would be worth undertaking further investigation as to how changes to the protocol and supporting information (particularly training resources) could enhance record quality. We did not capture information on the schools’ use of the available resources or the teachers’ existing knowledge, but we recommend that future projects seek to capture this information so the effectiveness of the resources can be evalued. Although we emphasised the importance of zero counts, we could have more strongly emphasised that we did not expect people to see many different colour types. Also subtle tweaks to the protocol could have had big effects, for example, asking which colour groups were *not* seen could provide more accurate information on the colour groups that *were* seen. Further careful research, including drawing on experience from psychology and sociology, will be valuable in designing future studies appropriate to the particular target audience, e.g. careful consideration of how to reduce bias and error by phrasing questions better.

In this project we sought to test a specific hypothesis which had been pre-defined by professional scientists. Hypothesis-led citizen science is a potentially powerful way of running citizen science because it engages people in exploring specific questions and not just relying on them as observers and recorders. The value of hypothesis-led citizen science is yet to be fully realised, although has been used previously to detect evolutionary change in snail banding patterns [44, 45] and the invasion dynamics of a leaf-mining moth [46]. Unfortunately due to limitations with the data we were unable to formally test our hypotheses about the impact of landscape at multiple scales on bumblebee colour group richness, otherwise this could have been compared with recent studies from professional scientists [47]. There is increasing emphasis on the importance of urban green spaces for biodiversity conservation [48], and so projects monitoring pollinators near to where participants live could result in greater engagement with local management, as is being proposed with the nascent Polli:Nation project in the UK (http://www.ltl.org.uk/pollination/). With the interest in urban management for nature [48] and ‘widlife-friendly gardening’ [49], volunteers could therefore play a vital role in gathering the important evidence-base to support management.

Previous research has indicated the importance of diversity, abundance and proximity of floral resources to the visitation activity of different flower visitor groups [50–52], although patch size itself has been shown not to affect visitation rate [53]. The Big Bumblebee Discovery demonstrated that the proximity to other flowers influenced the number of reported bumblebees visiting lavender; a higher rate was reported for lavender plants which were close to other flowers compared to lavender placed more than five metres from other flowers. This is similar to previous research showing that the local context affects visitation rate; e.g. flower-rich patches in flower-poor landscapes can preferentially attract insects [54]. The local abundance of flowers within urban landscapes is recognised as an important factor in determining bee species richness [47]. However, while wildflower strips in agricultural landscapes have been shown to enhance local bee abundance and richness [55], effects on bumblebees appear to increase with increasing landscape-wide floral resource availability [55]. Therefore, it is important to consider both local and landscape availability of floral resources when considering conservation management regimes aimed at promoting bumblebee populations [56]. Further research is required to elucidate how this complex interplay between local and landscape factors determines abundance and richness of species of pollinating insects ultimately impacts upon the ecosystem function of pollination.

In considering approaches for monitoring pollinators and pollination, we have shown that timed-counts of insects visiting a lure plant can be a way to engage large numbers of people in collecting monitoring data using a standardised methodology. However, there must be careful consideration of data accuracy and ensuring that the data are fit for purpose. Access to the established school programmes of both the British Science Association and EDF Energy, coupled with the high quality and attractive resources produced by these partner organisations in consultation with the authors, ensured the high level of participation in this one-year project. Promotion of the project was achieved through a diverse range of methods including on-line resources, national media campaigns and school visits. Future projects need to consider ways of recruiting participants and sustaining participation.

Given the high level of errors in the identification of colour groups, it would have been valuable to provide additional training resources, for example an on-line method of testing identification. If citizen science is to contribute the monitoring of pollinating insects then considerable resources must be invested in training volunteers even if a seemingly straightforward method such as colour groups [35] or broad taxonomic groups [34] of identification is employed. Also all participants could provide evidence of their observations (such as photographs) for verification [57]. Smartphones and tablets with in-built cameras allow users to submit high resolution photographs at the time of making the record [27]. This facilitates the submission of supporting evidence for verification of the observations; e.g. in the UK Ladybird Survey photos are submitted with 80% of reports via the smrtphone app, but only 40% via the website form [58, 59]. The verification of photographs can be time-consuming, but it is undoubtedly profitable in assuring high data quality and the process of verification can be used to provide feedback to participants, thus supporting their learning.

In conclusion, citizen science has considerable potential for undertaking research at spatial scales that would not otherwise be possible [4], but data quality remains key and should be considered carefully on a study-by-study basis, rather than simply relying on general evidence from previous studies. It is important to consider methods for ensuring accuracy (prior to data collection via training and supporting resources) and confirming this (after data collection via verification of submitted records) in order to ensure data quality is known and fit-for-purpose.

## Supporting Information

S1 Fig Log book. (PDF)

S2 Fig Posters. (PDF)

S3 Fig Factsheet. (PDF)

S4 Fig Experiment Guide. (PDF)

S1 Table Data. (CSV)

S2 Table Classification matrices of colour types of bumblebees. (DOCX)
