# Correction: Focal Plant Observations as a Standardised Method for Pollinator Monitoring: Opportunities and Limitations for Mass Participation Citizen Science

**DOI:** 10.1371/journal.pone.0155571

**Published:** 2016-05-09

**Authors:** Helen E. Roy, Elizabeth Baxter, Aoine Saunders, Michael J. O. Pocock

There is an error in the Methodology/Principal Findings subsection of the Abstract. The number of schools is incorrect. The correct sentence should read: Timed counts of bumblebees (*Bombus* spp.; identified to six colour groups) visiting focal plants of lavender (*Lavendula* spp.) were carried out by about 13 000 primary school children (7–11 years old) from over 400 schools across the UK. 3948 reports were received totaling 26 868 bumblebees.
