# Supplementary material for: Focal Plant Observations as a Standardised Method for Pollinator Monitoring: Opportunities and Limitations for Mass Participation Citizen Science
Source: PLoS One. 2016 Mar 17;11(3):e0150794. doi: 10.1371/journal.pone.0150794 (PMC4795797; doi:10.1371/journal.pone.0150794)
Supplement: S1 Fig — (PDF) [file pone.0150794.s001.pdf]

The Big BUMBLEBEE Discovery

Logbook

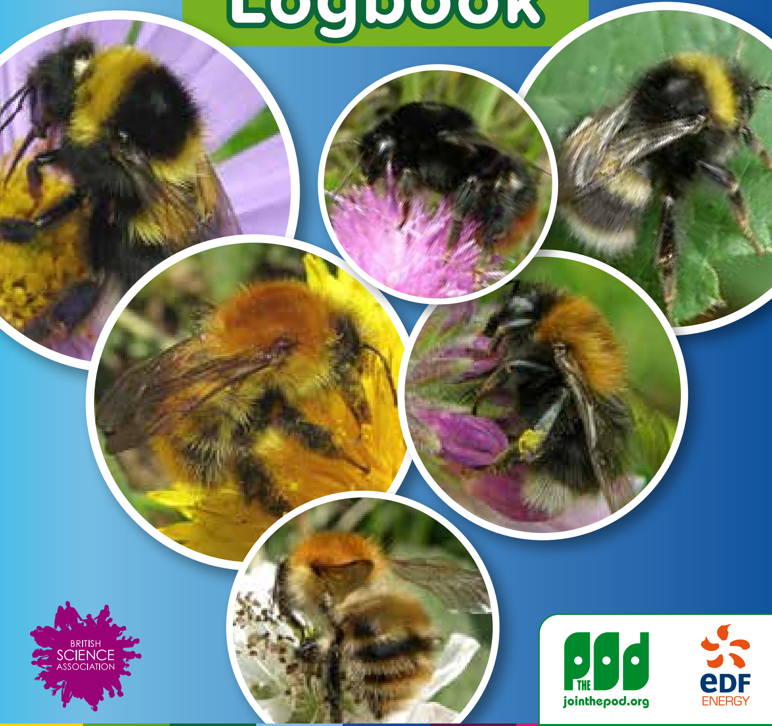

BRITISH SCIENCE ASSOCIATION

THE pod
jointhepod.org

EDF ENERGY

Energy | Science | Sustainability

THE BIG BUMBLEBEE DISCOVERY

Are you ready to become a citizen scientist this summer? By logging the number and colour group of bumblebees visiting lavender plants in the experiment, you'll be helping us to test how bumblebees are affected by their immediate and wider surroundings.

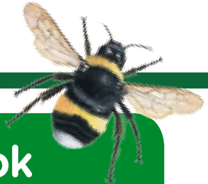

How to use this logbook

Use the identification notes on pages 4 to 9 to learn how to spot each of the six colour groups, then fill in pages 10-19 with your findings. Afterwards, visit **www.jointhepod.org/experiment-zone** to upload your data. It's really important you do this; otherwise your results won't count towards the scientific research.

When you upload your findings to the Pod, you'll need your blog code to make your observation count towards your schools' results.

Your school's blog code is ....

When you find a bumblebee from each colour group, put a sticker of it in the space next to it on the page.

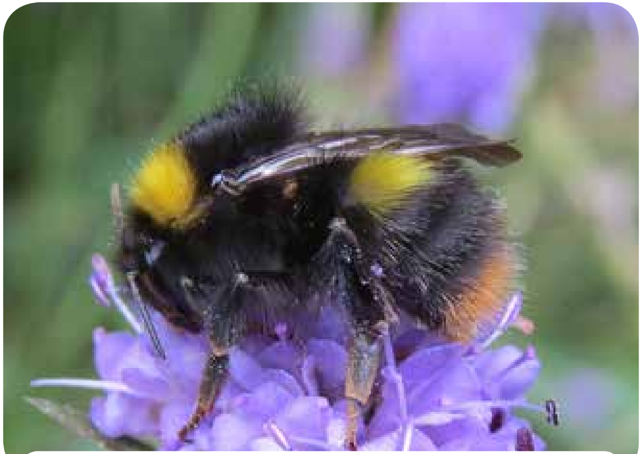

This logbook belongs to ....

CONTENTS

4 The **banded orange tail** colour group

6 The **black-bodied orange tail** colour group

6 The **two-banded white tail** colour group

7 The **three-banded white tail** colour group

8 The **brown** colour group

9 The **orange white tail** colour group

10 Notes for your observations

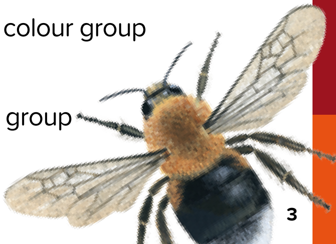

BANDED ORANGE TAIL

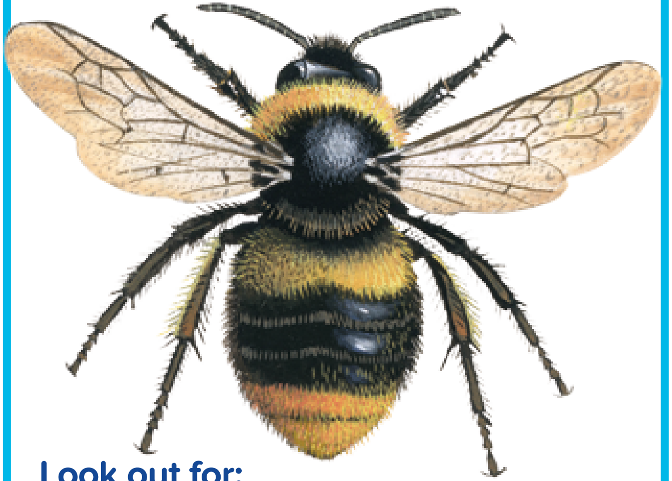

Look out for:

- [ ] A yellow stripe on the collar
- [ ] A yellow/orange stripe on the abdomen
- [ ] An orange/red tail tip

PLACE YOUR STICKER HERE

BUZZ FACT

Bumblebees in this colour group are often smaller than other bumblebees.

- [ ] Found this bumblebee?

BLACK-BODIED ORANGE TAIL

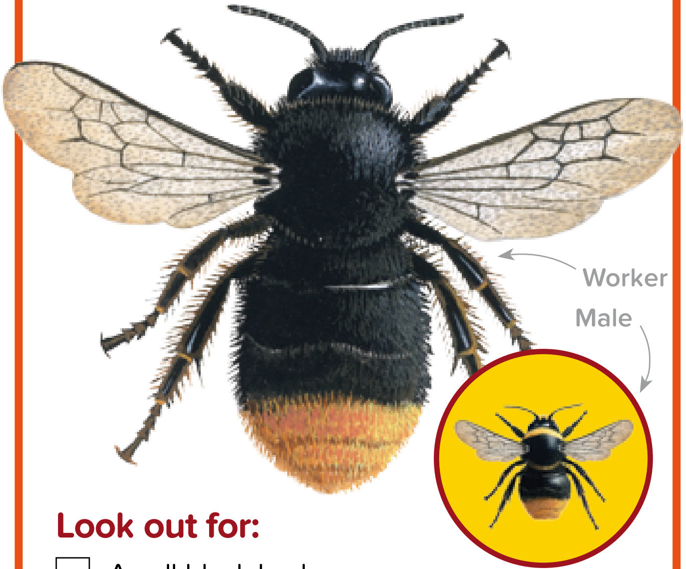

Look out for:

- [ ] An all-black body
- [ ] An orange/red tail

PLACE YOUR STICKER HERE

BUZZ FACT

If you spot one with a yellow strip on its thorax, it's a male rather than a queen or worker.

- [ ] Found this bumblebee?

TWO-BANDED WHITE TAIL

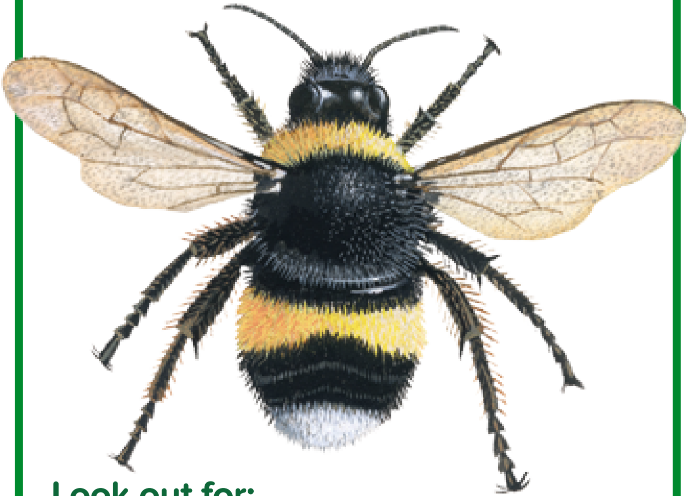

Look out for:

- [ ] **TWO** yellow stripes (one on the thorax and one on the abdomen)
- [ ] A white tail

PLACE YOUR STICKER HERE

BUZZ FACT

These bumblebees are very common; look for them wherever there are flowers with pollen!

- [ ] Found this bumblebee?

THREE-BANDED WHITE TAIL

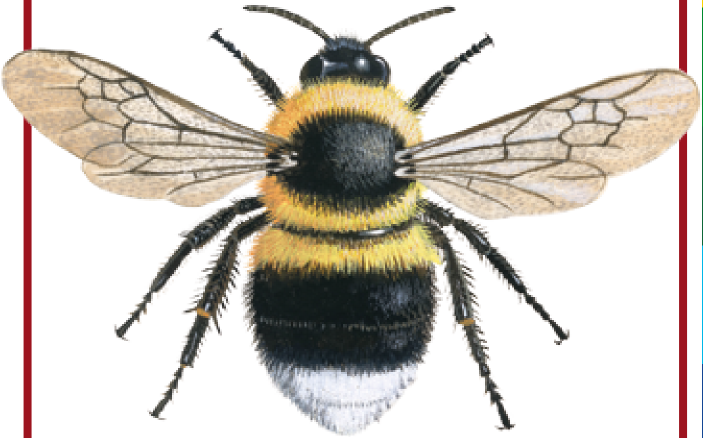

Look out for:

- [ ] **THREE** yellow stripes (two on the thorax and one on the abdomen)
- [ ] A white tail

BUZZ FACT

Many bumblebees in this colour group have very long tongues, so they love deep-tubed flowers, like lavender.

PLACE YOUR STICKER HERE

- [ ] Found this bumblebee?

BROWN

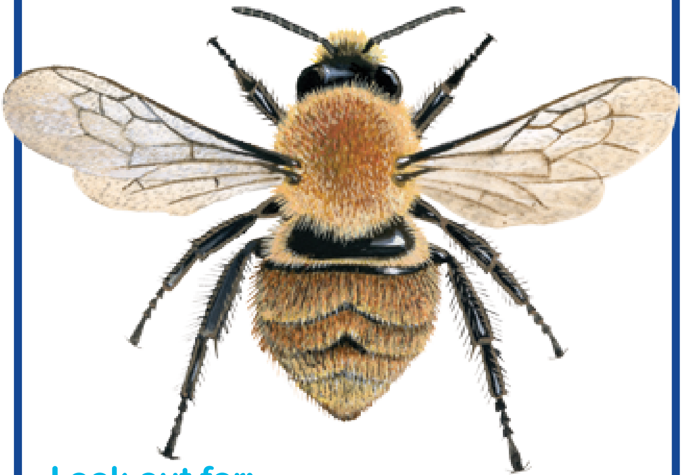

Look out for:

- ☐ Possible stripes on the bottom half, which get darker nearer the tail
- ☐ Sandy-brown/ginger thorax

PLACE YOUR STICKER HERE

BUZZ FACT

These bumblebees can be seen on flowers late in the year, even into November in southern Britain.

☐ Found this bumblebee?

ORANGE WHITE TAIL

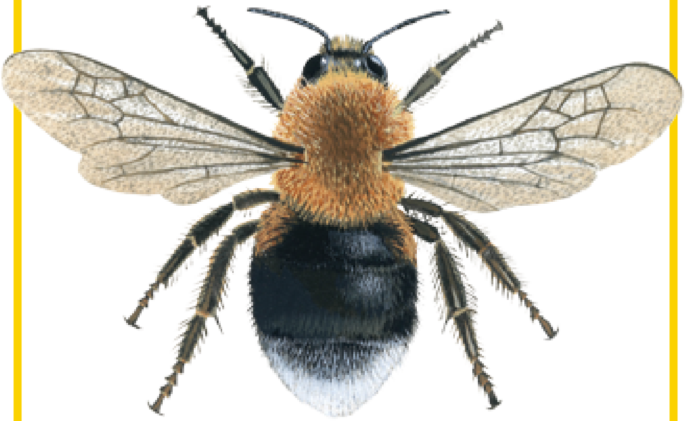

Look out for:

- ☐ Orange/brown thorax
- ☐ Black abdomen
- ☐ White tail

PLACE YOUR STICKER HERE

BUZZ FACT

This type of bumblebee only arrived here in 2001 from Europe, but has now spread as far north as Scotland.

☐ Found this bumblebee?

Observation 1

ABOUT YOUR LOCATION

1. Where are you counting your bumblebees? (e.g. York Road, Exeter)

2. Which best describes this location?

☐ In a town or city ☐ Suburban (e.g. village or edge of town)

☐ Green space within a town or city (e.g. school field or park)

☐ Countryside ☐ None of these

ABOUT YOUR EXPERIMENT

3. What is the date?

4. What time did you start? (Watch for at least 5 mins)

5. What time did you finish?

6. What is the weather like? (Bumblebees don't like cold, wet weather!)

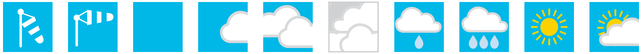

☐	☐	☐	☐	☐	☐	☐	☐	☐	☐
Still/slight breeze	Windy	No cloud	Less than ½ cloud cover	More than ½ cloud cover	Complete cover	Light rain	Heavy rain	Bright sun	Sunny intervals

ABOUT YOUR LAVENDER*

7. What lavender are you using?

☐ A Big Bumblebee Discovery pot of lavender from school

☐ Your own pot ☐ A patch of lavender

*Only use lavender that's in flower for the experiment.

8. What type of lavender are you observing?

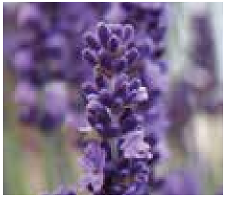

☐ English

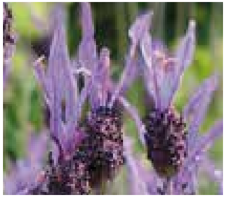

☐ French

9. How big is the area of lavender? (Max width and length in cms)

10. Which best describes the position of your lavender?

☐ Less than 30cm from the other flowers (length of A4 paper)

☐ Less than 1m from other flowers (around 2 paces)

☐ Less than 5m from other flowers (around 10 paces)

☐ More than 5m from other flowers (10 paces away)

ABOUT YOUR DISCOVERY

11. How many bumblebees of each colour group did you see?

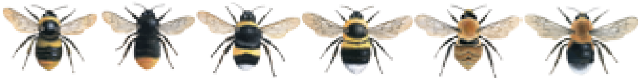

☐	☐	☐	☐	☐	☐
Banded orange tail	Black-bodied orange tail	Two-banded white tail	Three-banded white tail	Brown	Orange white tail

☐ I've entered my data on the Pod

Observation 2

ABOUT YOUR LOCATION

1. Where are you counting your bumblebees? (e.g. York Road, Exeter)

2. Which best describes this location?

☐ In a town or city ☐ Suburban (e.g. village or edge of town)

☐ Green space within a town or city (e.g. school field or park)

☐ Countryside ☐ None of these

ABOUT YOUR EXPERIMENT

3. What is the date?

4. What time did you start? (Watch for at least 5 mins)

5. What time did you finish?

6. What is the weather like? (Bumblebees don't like cold, wet weather!)

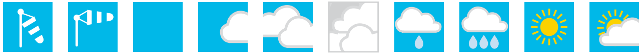

☐ Still/slight breeze ☐ Windy ☐ No cloud ☐ Less than ½ cloud cover ☐ More than ½ cloud cover ☐ Complete cover ☐ Light rain ☐ Heavy rain ☐ Bright sun ☐ Sunny intervals

ABOUT YOUR LAVENDER*

7. What lavender are you using?

☐ A Big Bumblebee Discovery pot of lavender from school

☐ Your own pot ☐ A patch of lavender

*Only use lavender that's in flower for the experiment.

8. What type of lavender are you observing?

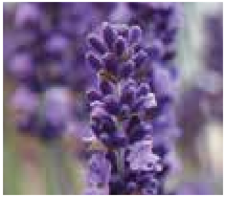

☐ English

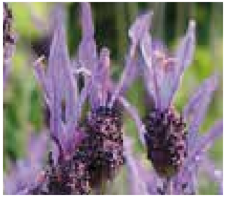

☐ French

9. How big is the area of lavender? (Max width and length in cms)

10. Which best describes the position of your lavender?

☐ Less than 30cm from the other flowers (length of A4 paper)

☐ Less than 1m from other flowers (around 2 paces)

☐ Less than 5m from other flowers (around 10 paces)

☐ More than 5m from other flowers (10 paces away)

ABOUT YOUR DISCOVERY

11. How many bumblebees of each colour group did you see?

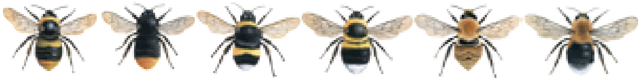

☐ Banded orange tail ☐ Black-bodied orange tail ☐ Two-banded white tail ☐ Three-banded white tail ☐ Brown ☐ Orange white tail

☐ I've entered my data on the Pod

Observation 3

ABOUT YOUR LOCATION

1. Where are you counting your bumblebees? (e.g. York Road, Exeter)

2. Which best describes this location?

☐ In a town or city ☐ Suburban (e.g. village or edge of town)

☐ Green space within a town or city (e.g. school field or park)

☐ Countryside ☐ None of these

ABOUT YOUR EXPERIMENT

3. What is the date?

4. What time did you start? (Watch for at least 5 mins)

5. What time did you finish?

6. What is the weather like? (Bumblebees don't like cold, wet weather!)

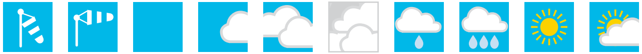

☐	☐	☐	☐	☐	☐	☐	☐	☐	☐
Still/slight breeze	Windy	No cloud	Less than ½ cloud cover	More than ½ cloud cover	Complete cover	Light rain	Heavy rain	Bright sun	Sunny intervals

ABOUT YOUR LAVENDER*

7. What lavender are you using?

☐ A Big Bumblebee Discovery pot of lavender from school

☐ Your own pot ☐ A patch of lavender

*Only use lavender that's in flower for the experiment.

8. What type of lavender are you observing?

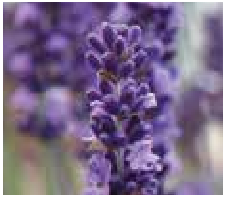

☐ English

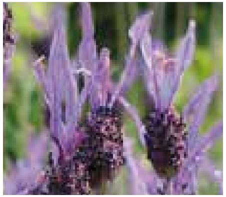

☐ French

9. How big is the area of lavender? (Max width and length in cms)

10. Which best describes the position of your lavender?

☐ Less than 30cm from the other flowers (length of A4 paper)

☐ Less than 1m from other flowers (around 2 paces)

☐ Less than 5m from other flowers (around 10 paces)

☐ More than 5m from other flowers (10 paces away)

ABOUT YOUR DISCOVERY

11. How many bumblebees of each colour group did you see?

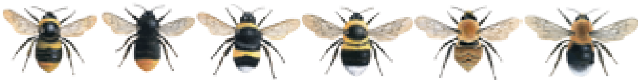

☐	☐	☐	☐	☐	☐
Banded orange tail	Black-bodied orange tail	Two-banded white tail	Three-banded white tail	Brown	Orange white tail

Filled up your logbook? Well done!

Download more tracking sheets to fill in from the Pod.

☐ I've entered my data on the Pod

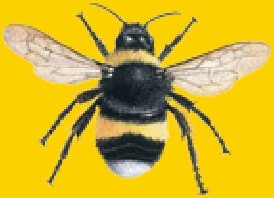

DON'T FORGET...

It's really important you upload your results after each observation. The input of every citizen scientist – student, teacher, parent and community group – is critical to the experiment, and vital in helping us to secure the future of our bumblebee population.

Upload your results at:
www.jointhepod.org/experiment-zone

With thanks to **Dr Michael Pocock** and **Dr Helen Roy** from the Centre for Ecology & Hydrology.

Other sources:
www.wildlifetrusts.org/species/white-tailed-bumblebee
www.bbc.co.uk/news/uk-scotland-glasgow-west-23062663
www.nhm.ac.uk/nature-online/life/insects-spiders/identification-guides-and-keys/bee-rotate/carder-bee/index.html

EDF Energy is a trading name used by EDF Energy Customers plc
(Reg.No. 02228297, registered office 40 Grosvenor Place, London SW1X 7EN).
