# Supplementary material for: Focal Plant Observations as a Standardised Method for Pollinator Monitoring: Opportunities and Limitations for Mass Participation Citizen Science
Source: PLoS One. 2016 Mar 17;11(3):e0150794. doi: 10.1371/journal.pone.0150794 (PMC4795797; doi:10.1371/journal.pone.0150794)
Supplement: S2 Fig — (PDF) [file pone.0150794.s002.pdf]

The Big BUMBLEBEE Discovery

EARLY BUMBLEBEE

Bombus pratorum

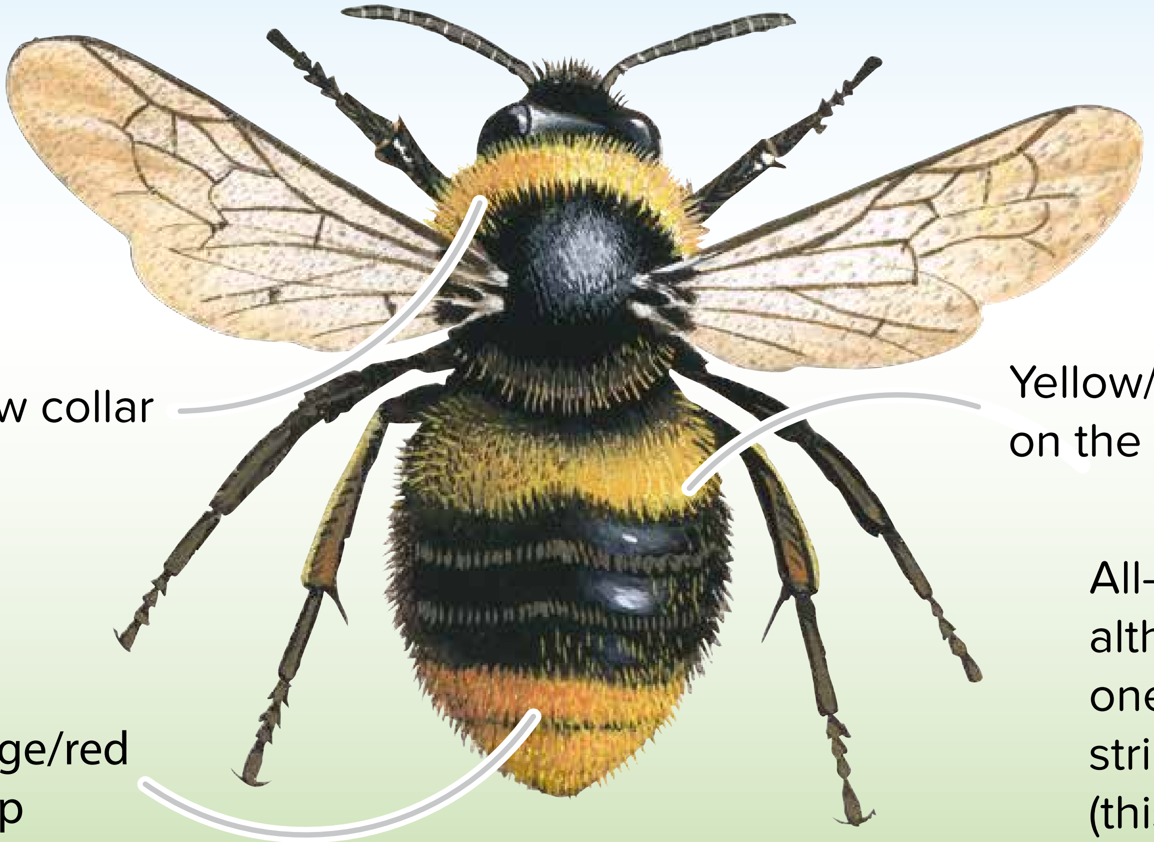

Yellow collar

Yellow/orange stripe on the abdomen

Orange/red tail tip

Colour group: Banded orange tail

RED-TAILED BUMBLEBEE

Bombus lapidarius

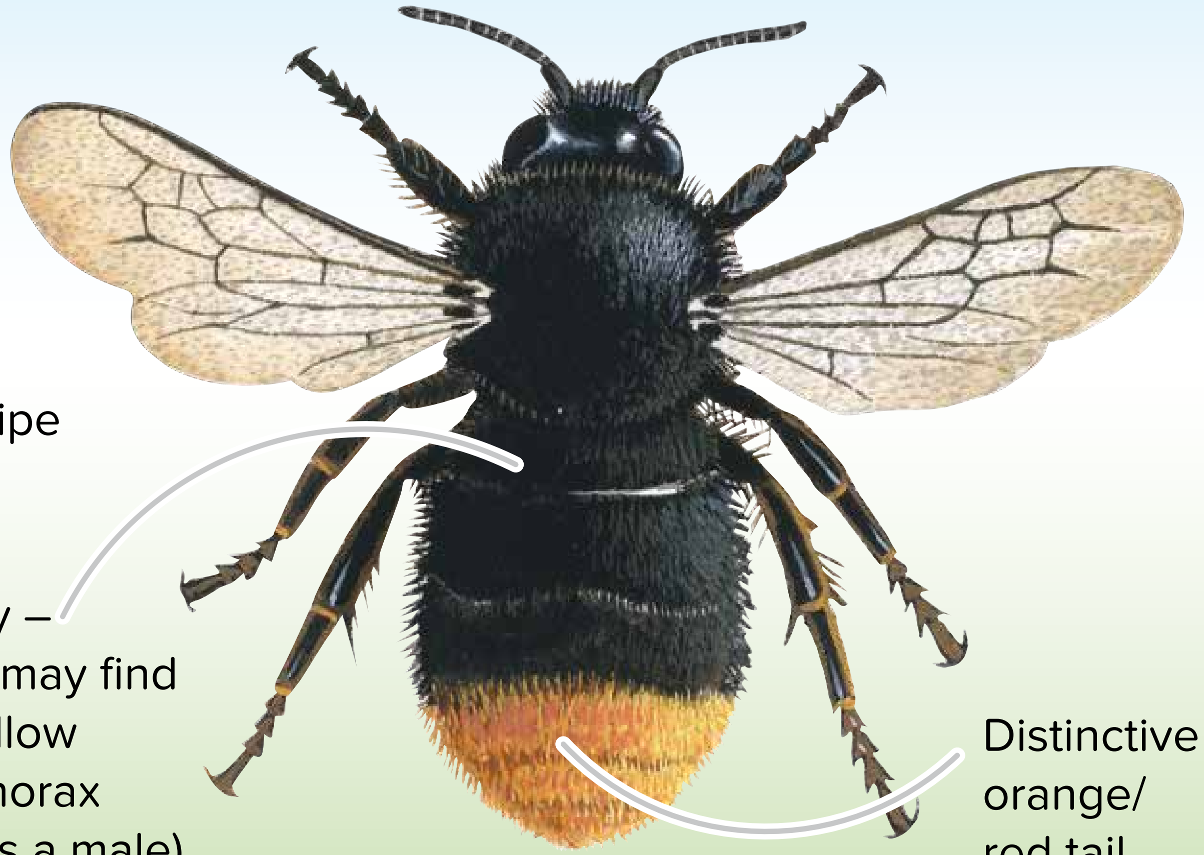

All-black body – although you may find one with a yellow stripe on its thorax (this means it's a male)

Distinctive orange/red tail

Colour group: Black-bodied orange tail

BUZZZZ... DID YOU KNOW?

The early bumblebee is smaller than other bumblebees, so it can get in hard-to-reach plants, upside-down or drooping flowers. The Red-Tailed bumblebee is bigger, so it prefers to land on flowers with a 'platform', like a daisy.

Scientists use Latin names to identify different bumblebees – and the same names are used all over the world. The first word describes the 'family' it belongs to, so *bombus* means bumblebee.

BRITISH SCIENCE ASSOCIATION

THE pod jointhepod.org

EDF ENERGY

Energy | Science | Sustainability

With thanks to Dr Michael Pocock and Dr Helen Roy from the Centre for Ecology & Hydrology. Other sources include: www.helpsavebees.co.uk/bumblebee-ID-chart.html; Bumblebee Conservation Trust; www.wildaboutgardens.org.uk /thingstodo/allyearround/flowers-for-bees.aspx
EDF Energy is a trading name used by EDF Energy Customers plc (Reg.No. 02228297, registered office 40 Grosvenor Place, London SW1X 7EN)

The Big
BUMBLEBEE Discovery

COMMON BUMBLEBEE
Bombus lucorum

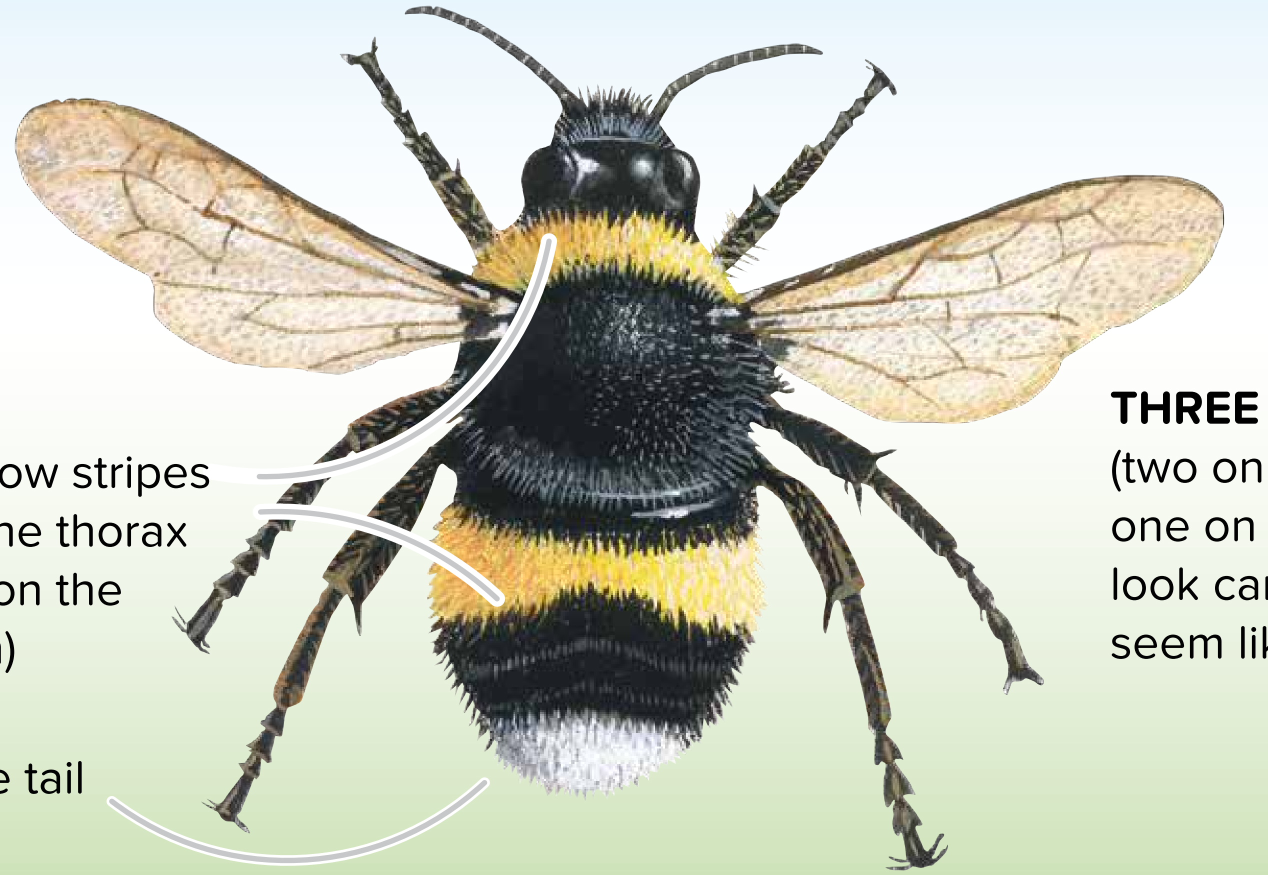

TWO yellow stripes (one on the thorax and one on the abdomen)

White tail

Colour group: Two-banded white tail

GARDEN BUMBLEBEE
Bombus hortorum

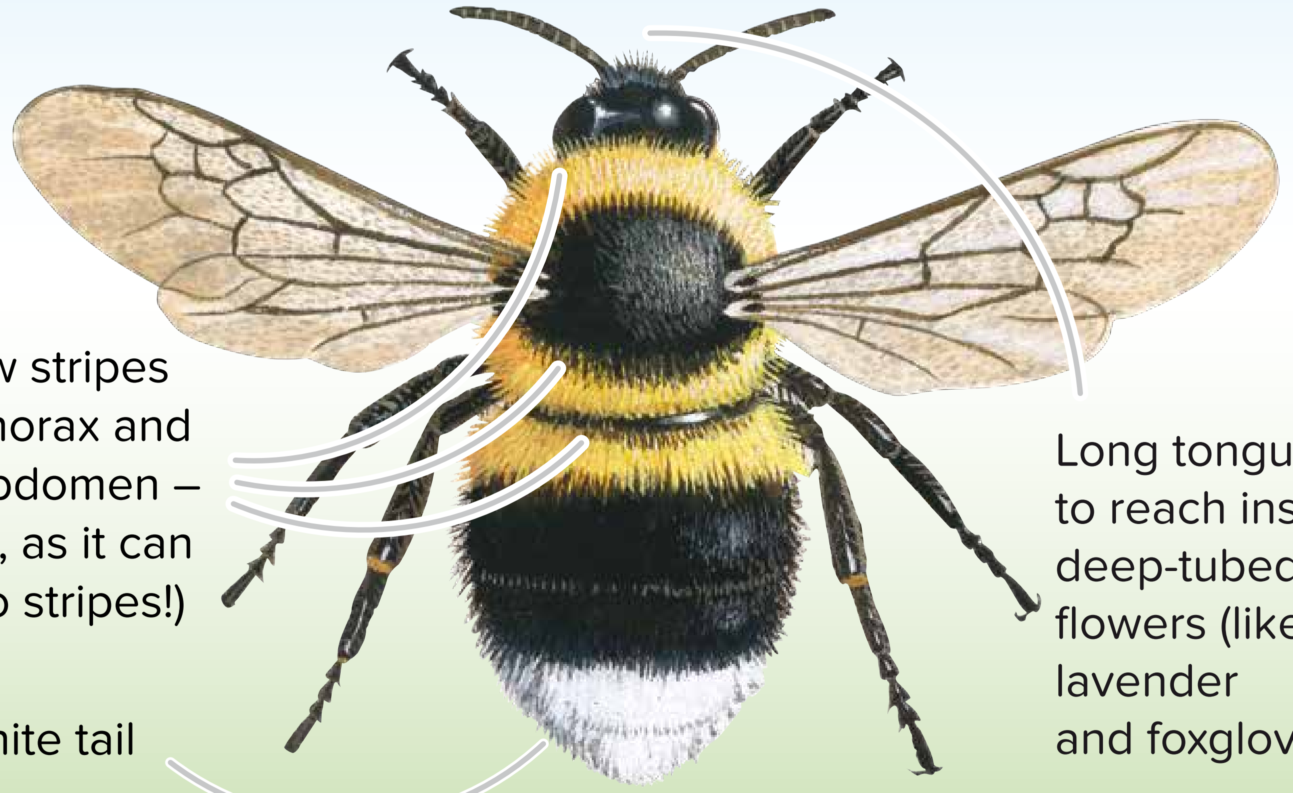

THREE yellow stripes (two on the thorax and one on the abdomen – look carefully, as it can seem like two stripes!)

Long tongue to reach inside deep-tubed flowers (like lavender and foxgloves)

White tail

Colour group: Three-banded white tail

BUZZZZ... DID YOU KNOW?

Bumblebee tongues can be as long as 9mm! It makes them very efficient at extracting nectar from the tubular flowers of lavender.

The common bumblebee can be what's known as a 'nectar robber'. This means it bites a hole in the flower to reach the nectar.

Sources: With thanks to Dr Michael Pocock and Dr Helen Roy from the Centre for Ecology & Hydrology. Other sources include: www.wildaboutgardens.org.uk; www.bto.org/sites/default/files/u23/downloads/pdfs/bbees-645kb.pdf; 'Longer tongues and swifter handling' by Balfour, N. J of the University of Sussex.
EDF Energy is a trading name used by EDF Energy Customers plc (Reg.No. 02228297, registered office 40 Grosvenor Place, London SW1X 7EN)

BRITISH SCIENCE ASSOCIATION

THE pod
jointhepod.org

EDF ENERGY

Energy | Science | Sustainability

The Big BUMBLEBEE Discovery

CARDER BUMBLEBEE

Bombus pascuorum

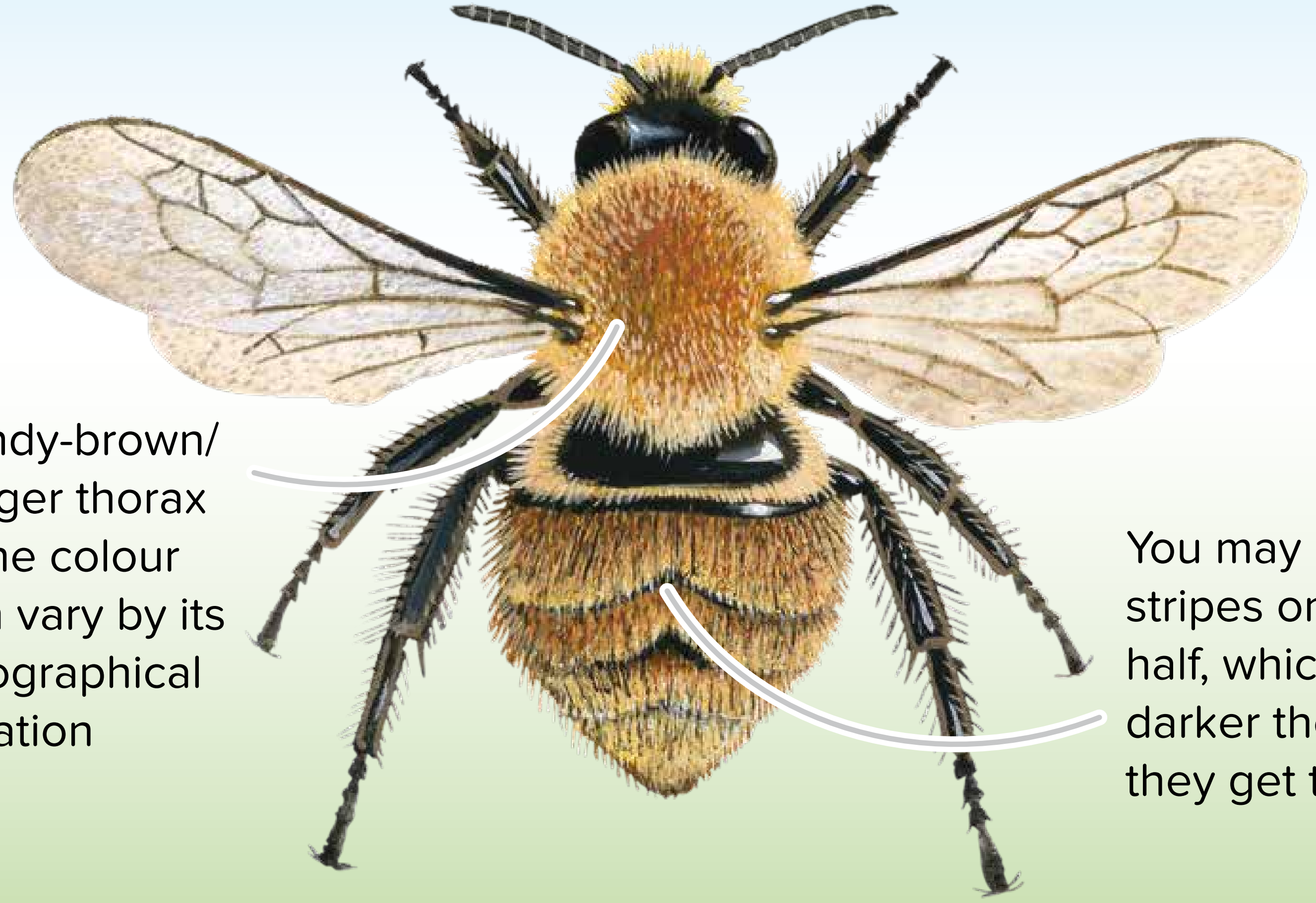

Colour group: Brown

TREE BUMBLEBEE

Bombus hypnorum

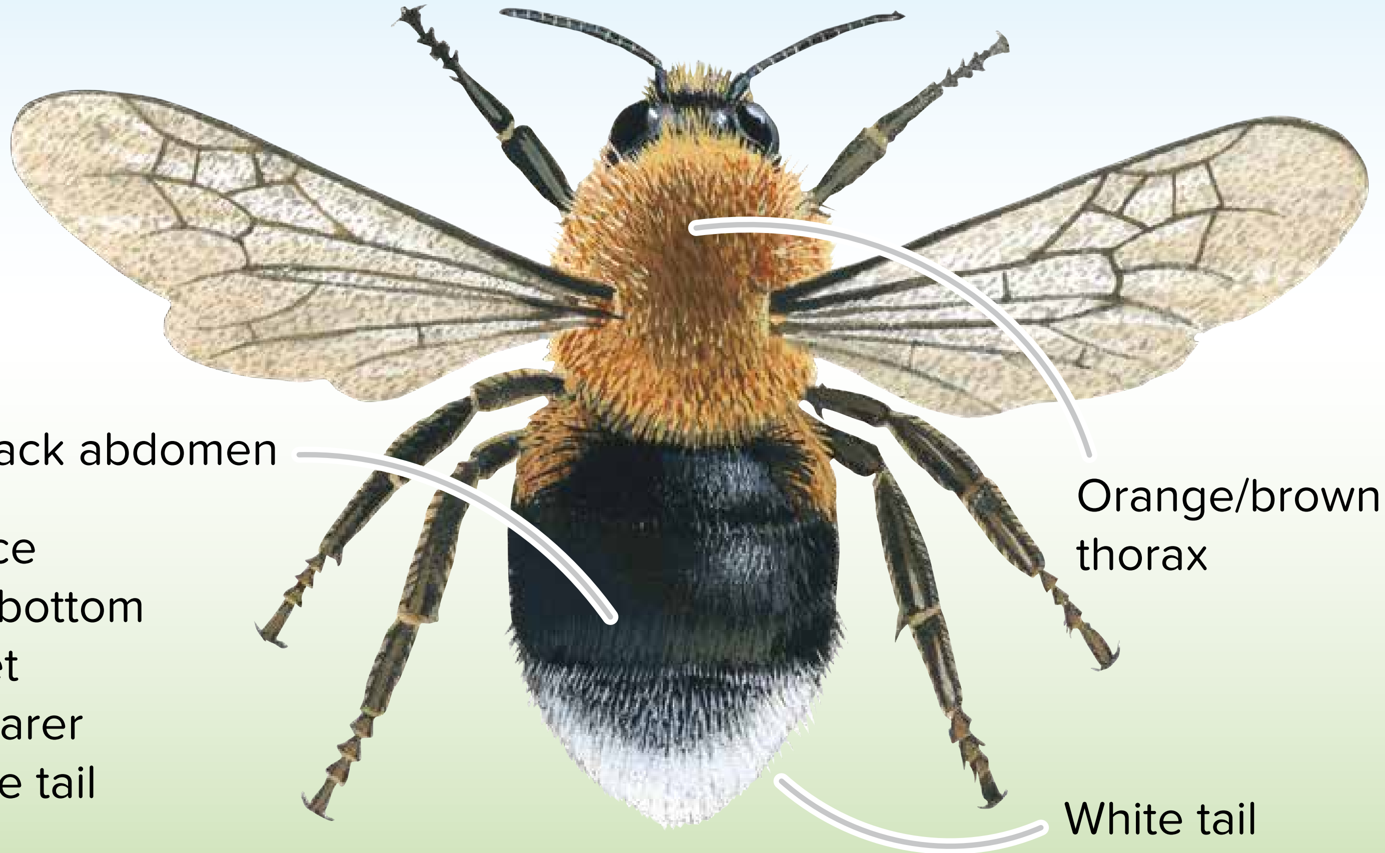

Colour group: Orange white tail

BUZZZZ... DID YOU KNOW?

- The carder bumblebee can be found on flowers as late in the year as November in southern Britain.
- The tree bumblebee is the only British bumblebee to nest above ground level. It nests in tree holes, bird boxes and even holes in roofs.

Sources: With thanks to Dr Michael Pocock and Dr Helen Roy from the Centre for Ecology & Hydrology.
Other sources include: www.helpsavebees.co.uk/ bumblebee-ID-chart.html; Bumblebee Conservation Trust.
EDF Energy is a trading name used by EDF Energy Customers plc (Reg.No. 02228297, registered office 40 Grosvenor Place, London SW1X 7EN)

BRITISH SCIENCE ASSOCIATION
THE pod
jointhepod.org
EDF ENERGY
Energy | Science | Sustainability
