# Supplementary material for: Focal Plant Observations as a Standardised Method for Pollinator Monitoring: Opportunities and Limitations for Mass Participation Citizen Science
Source: PLoS One. 2016 Mar 17;11(3):e0150794. doi: 10.1371/journal.pone.0150794 (PMC4795797; doi:10.1371/journal.pone.0150794)
Supplement: S3 Fig — (PDF) [file pone.0150794.s003.pdf]

The Big BUMBLEBEE Discovery

Things you may not know about Bumblebees

Pollination is when pollen is taken from one plant to another by a pollinator.

Pollination is very important for our environment. It helps plants to produce seed, so more can grow.

Bumblebees can move their wings up to 200 times per second. This produces the 'buzzing' sounds as they fly.

Bumblebees like flowers that have lots of pollen and nectar, such as lavender, fuschias, foxgloves, geraniums, rosemary and other herbs.

Bumblebees are a type of pollinator. Other insects that act as pollinators include hoverflies, butterflies and moths.

Plants can also be pollinated by wind and water.

In each nest there are different types of bumblebee – a queen, female workers and males. All of the workers are sisters to each other, so when they bring back pollen to feed the young, they are helping to raise their sisters.

Bumblebees have a special way of pollinating called buzz pollination, and no other pollinator can do it! Some flowers trap their pollen, but it can be released if a bumblebee lands on it and buzzes in just the right way – this is how tomatoes are pollinated.

Bumblebees are specially designed to gather pollen. They have long tongues so they can reach into tube-shaped flowers and drink the nectar. They also have hairy bodies and special "baskets" on their back legs which can carry pollen.

Queen bumblebees live for one year, but workers and males only live for a few months.

Bumblebees form nests with 50-400 bees!

There are 24 different species of bumblebee in the UK and all but the rarest fit into one of six colour types (we'll be looking at them in the Big Bumblebee Discovery!)

To make sure that other bumblebees don't visit a flower and find all of the nectar is gone, bumblebees leave their scent to tell other bees not to waste their time!

British Science Association

This information has been sourced from the Centre for Ecology and Hydrology, the Bumblebee Conservation Trust and www.parliament.uk.

The pod
jointhepod.org

EDF Energy

Energy | Science | Sustainability
