# Supplementary material for: Focal Plant Observations as a Standardised Method for Pollinator Monitoring: Opportunities and Limitations for Mass Participation Citizen Science
Source: PLoS One. 2016 Mar 17;11(3):e0150794. doi: 10.1371/journal.pone.0150794 (PMC4795797; doi:10.1371/journal.pone.0150794)
Supplement: S4 Fig — (PDF) [file pone.0150794.s004.pdf]

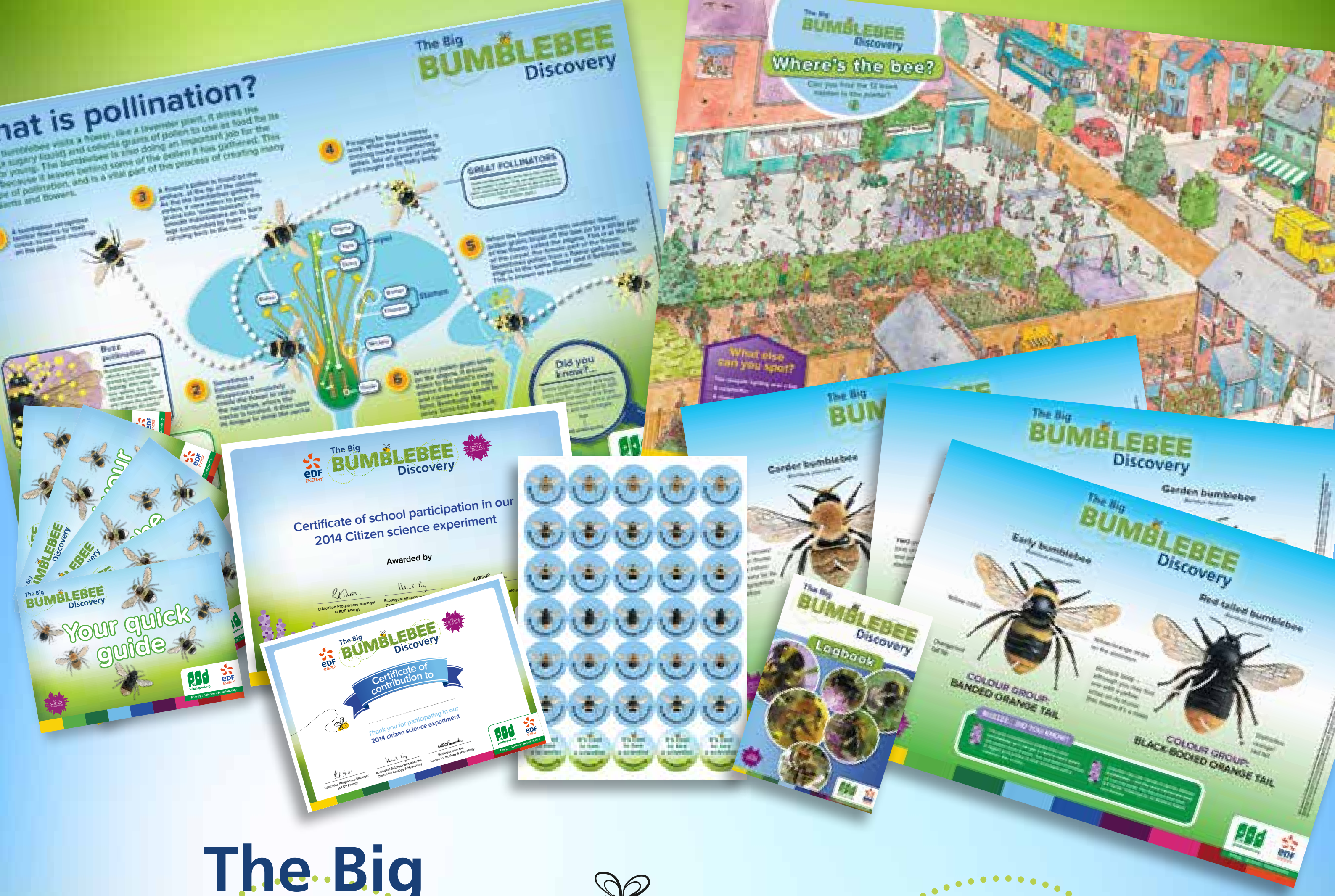

The Big BUMBLEBEE Discovery

Experiment Guide

BRITISH SCIENCE ASSOCIATION

THE POD
jointhepod.org

EDF ENERGY

Energy | Science | Sustainability

Contents

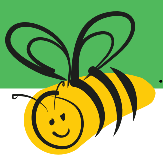

LEARN

p3 About The Big Bumblebee Discovery
p4 Your experiment checklist
p5 Sign up to the experiment
p6 Free experiment resources

ACT

p7 Run your experiment
p8 Record your observations

INSPIRE

p9 Encourage families, friends and community groups to join in
p10 Inspire tomorrow's scientists
p11 Useful resources

THINK!

The Big Bumblebee Discovery is an opportunity to engage students in the wonders of scientific discovery. Throughout the guide, look out for these Think! sections, which give ideas for things students can investigate on their own.

jointhepod.org

EDF ENERGY

Energy | Science | Sustainability

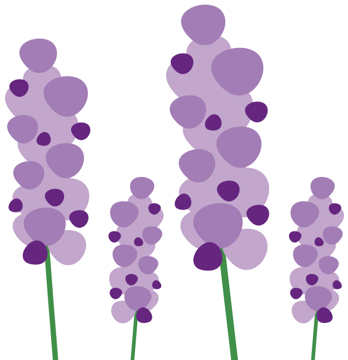

About The Big Bumblebee Discovery

Find out what the experiment is all about and why you won't want to miss out

One of the main aims of the Pod is to inspire a generation of young people to engage with STEM subjects (in particular science) and ultimately, help solve the energy challenges of the future. To help build on the successes of this programme we've launched The Great EDF Energy Experiment – a five-year programme of citizen science experiments that will make science accessible and open to as many people as possible.

The Great EDF Energy Experiment

The Big Bumblebee Discovery

Our first experiment is The Big Bumblebee Discovery. This is an opportunity to engage your students in citizen science this summer and open their minds to the fun they can have in running experiments. We've teamed up with the British Science Association and Centre for Ecology & Hydrology to gather data and investigate the diversity of bumblebees in the UK. We want schools to join in and help test the hypothesis that the diversity of bumblebees is affected by their immediate surroundings and the wider landscape.

The experiment

It's easy to take part – and you can run the experiment as often as you like between the beginning of June and the end of August. If you're one of the first 3,000 schools to sign up to The Big Bumblebee Discovery, you'll receive a free lavender plant and engagement pack. All your students need to do is spend at least five minutes observing how many bumblebees visit the lavender and what colour group they represent (see page 5 for more details). Afterwards, they need to upload this data to the Pod so it can be used in the scientific research. It's as simple as that!

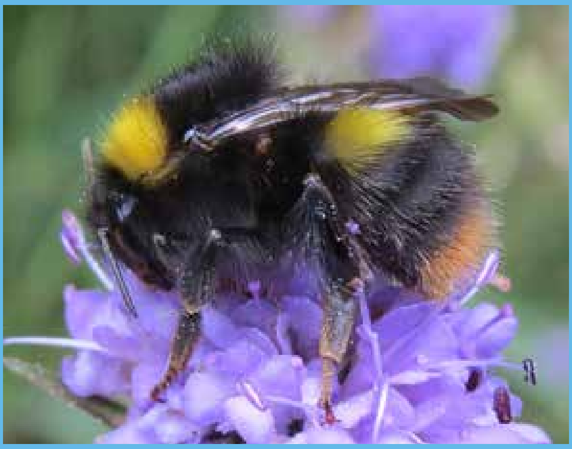

WHY BUMBLEBEES AND LAVENDER?

Bumblebees are excellent pollinators, but many people are extremely worried that the number of bumblebees is declining. **The Big Bumblebee Discovery** is a chance for your students to collect data that could help us understand more about these insects – and potentially prevent a decline in their numbers. We're using lavender because bumblebees love visiting lavender flowers, and lavender is flowering when we are running the experiment (June to August).

"Our goal is to inspire a generation of scientists and to **recruit 100,000 children** and their parents to participate in a series of live, breakthrough mass participation experiments over the next five years – all of which address **real scientific questions** about the impact of climate and environmental change in the UK."

jointhepod.org

Energy | Science | Sustainability

About The Big Bumblebee Discovery

WHAT IS CITIZEN SCIENCE?

Citizen science is the involvement of volunteers in science. By encouraging a little effort from a large number of individuals, data can be collected quickly and efficiently to help answer scientific questions. It's a method that scientists have been using for many years to investigate the natural world.Find out more in the **introductory video**.

Who we're working with

We've teamed up with Dr Michael Pocock and Dr Helen Roy from the Centre of Ecology & Hydrology for this experiment. Both are distinguished ecologists who are passionate about citizen science.

Find out more at www.ceh.ac.uk

Centre for Ecology & Hydrology
NATURAL ENVIRONMENT RESEARCH COUNCIL

> The input of every **citizen scientist** is critical to the experiment, and vital in helping us **secure the future** of our bumblebee populations.

Experiment checklist

Learn

- [ ] **Sign up** to The Big Bumblebee Discovery in the **Experiment Zone** on the Pod.
- [ ] Download some free **experiment resources** for teachers and students.
- [ ] If you're one of the first 3,000 schools to sign up and receive a free engagement pack and lavender plant, decide where to plant your lavender – or keep it in the pot.

Act

- [ ] Run the experiment as often as you want!
- [ ] Upload your findings to **www.jointhepod.org/experiment-zone/**.
- [ ] **Blog on the Pod** about your observations – it's a great way to share what you're doing with parents, schools and groups.

Inspire

- [ ] Use the stickers and certificates to reward your students.
- [ ] Encourage everyone to continue observations over the summer holidays.
- [ ] Hand out the logbooks and tracking charts, and signpost parents to the **discovery pack** for more information.

THINK!

Ask your students: Think about what would happen if pollinating insects disappeared – how might this impact on us? How could we encourage growing numbers of pollinators? What affects the numbers of pollinators?

THE pod
jointhepod.org
EDF ENERGY
Energy | Science | Sustainability

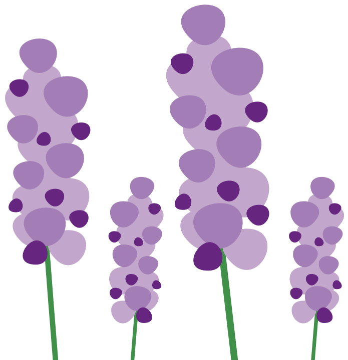

LEARN

Sign up to the experiment

Find out how to take part in The Big Bumblebee Discovery

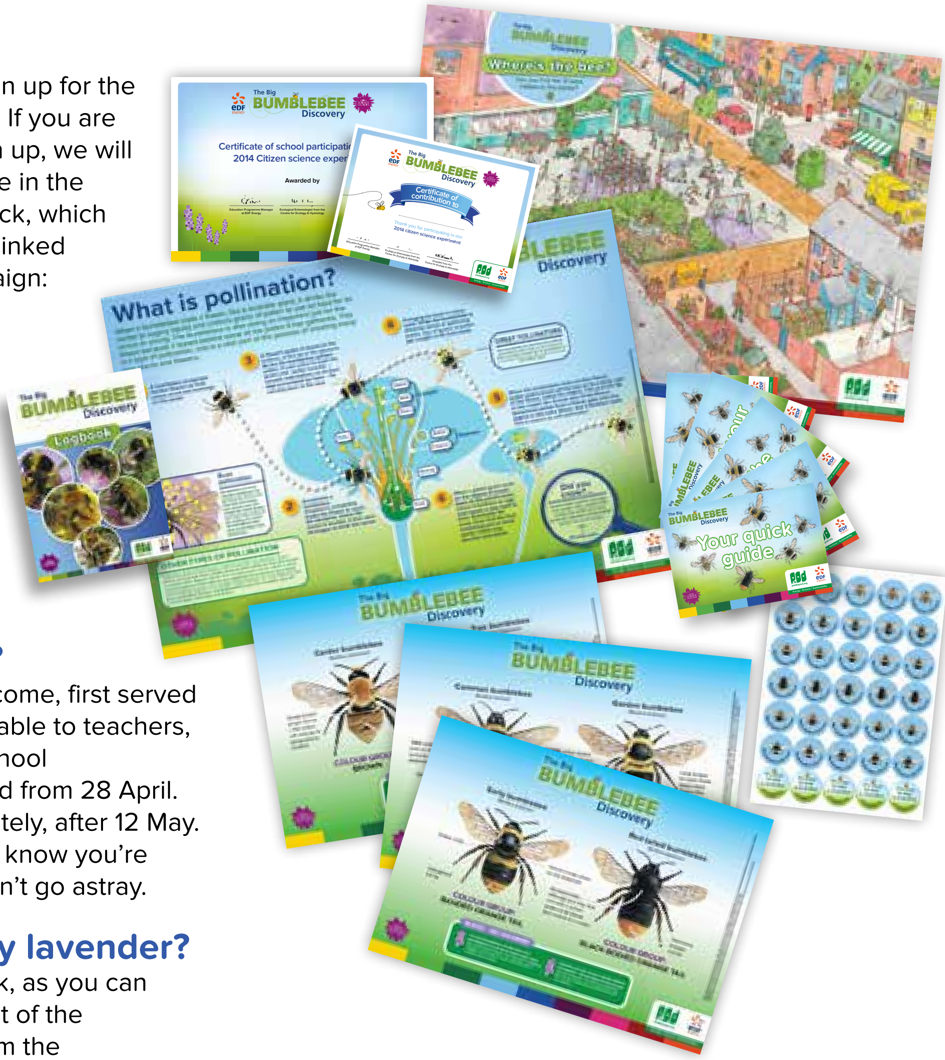

How do I sign up?

Register or log in on the Pod and sign up for the experiment in the **Experiment Zone**. If you are one of the first 3,000 schools to sign up, we will send you a free lavender plant to use in the experiment, plus an engagement pack, which contains lots of fantastic curriculum-linked resources to help you run the campaign:

- **Bee identification posters**
- **Poster about pollination**
- **Where's the bee poster**
- **Instruction cards**
- **Stickers**
- **Logbooks**
- **School certificate**
- **Student certificates**
- **Recycled pencils**

Can anybody get a pack?

The packs are distributed on a first come, first served basis (one per school); and are available to teachers, teaching assistants, governors or school administrators. They will be delivered from 28 April. The lavender plant will arrive separately, after 12 May. Don't forget to let your school office know you're expecting two deliveries, so they don't go astray.

What if we don't have any lavender?

Don't worry if you miss out on a pack, as you can still take part in the experiment. Most of the content is available to download from the **Experiment Zone**, and you can use your own lavender plant or observe lavender in a public place (a local park, for example).

When should I run the experiment?

You can carry out the bumblebee observations at any point between the beginning of June and end of August – and as often as you want. In fact, we want you to do as many experiments as you can, so we can build up a more complete picture of bumblebee diversity in the UK.

THINK!

Ask your students: Think about food chains that rely on pollinators. How many can you name and what would happen if we didn't have bumblebees for pollination?

Still got a question?

Email us at contact_us@jointhepod.org

jointhepod.org

EDF ENERGY

Energy | Science | Sustainability

LEARN

Free experiment resources

Put the experiment in context with resources to enrich students' learning

Background context

There are lots of curriculum-linked resources on the Pod to bring the topic to life for students and put the experiment in the context of their learning.

POD TOP TIPS:

- Why not kick off the experiment with a **whole school assembly on pollination**?
- You could create a display using the **posters** in your pack.
- The **introductory video** is a fun way of introducing the experiment to everyone.

Resources for the classroom

There are several classroom resources to help students understand more about the importance of bumblebees in pollinating flowers, fruit and vegetables.

POD TOP TIPS:

- Prepare with lessons on pollination and citizen science using the **lesson plans**. Both can be adapted to suit the ages and abilities of your students.
- Download the **pollination information pack** to swot up on key pollination facts. It can also be used by older children for their own research.

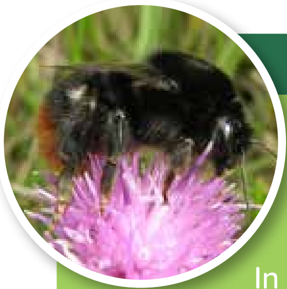

Did you know?

There are 24 different species of bumblebee in the UK and these fit into six different colour groups. In the experiment, students will be recording how many bees they spot from each colour group, rather than having to identify them as each particular species.

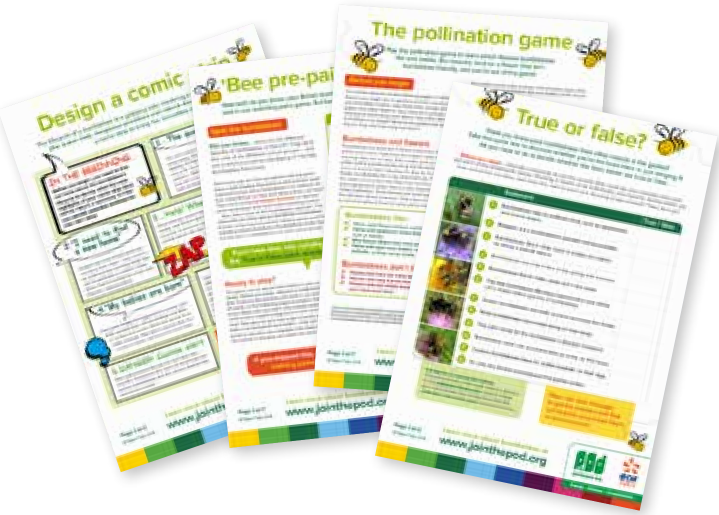

Bumblebee-inspired activities

There are lots of bumblebee-related resources on the Pod to help students identify the different colour groups.

POD TOP TIPS:

- Play a lively and fast-paced round of matching pairs in the **'bee pre-paired' training game** (there's also a **downloadable card version** of the game to play offline).
- Download the bumblebee identification chart and hand it out to students.
- Stick the **bumblebee identification posters** on the wall.
- Test how much information students have retained with **The Pollination Game** (for 4-7s), **True or False** (7-14s) or **Design a Comic Strip** (7-14s) quick activities.
- For a fun five-minute exercise, see if students can spot all 12 bumblebees in the **Where's the bee poster**.

THINK!

Ask your students: Why do you think plants are often brightly coloured and scented?

THE Pod jointhepod.org

EDF ENERGY

Energy | Science | Sustainability

ACT

Run your experiment

Here are the Pod's top tips for getting the most out of the experiment

BEFORE YOU BEGIN

Plan ahead so you're prepared on the day:

Choose your lavender

You can use *any* lavender for the experiment; and if you're using the pot we send you, it's up to you whether you plant it or keep it in the pot. If you do decide to plant it, consider which spots might offer the best opportunities for attracting bumblebees (e.g. where's the sunniest location?). If you are keeping the pot indoors, remember to put it outside for ten minutes before you run the experiment to attract insects.

Pick the right conditions for running your experiment

The best time to run your experiment is between mid-morning and mid-afternoon, when it's warmest. Insects prefer dry, sunny weather so if it's cold or damp outside, you might want to postpone the experiment for another day, as you won't spot many bumblebees.

Remember: you can carry out as many bumblebee observations as you want between **June and August**. So if the students enjoy it – or perhaps don't see many bumblebees on one day – plan another observation period!

"Inspiring young children through programmes like this can ensure we have good scientists and engineers, and find hidden talent that will change the world."

Saheefa Ishaq
14-year-old CREST award winner

WHAT IF WE DON'T SPOT ANY BUMBLEBEES?

It's unlikely that you'll spot all six colour groups – and you might be unlucky enough not to see any bumblebees. If this happens, upload your findings as they're still important and reschedule the experiment for another day or a different time. You may also want to move the lavender to a sunnier location or nearer to other flowers, if it isn't already.

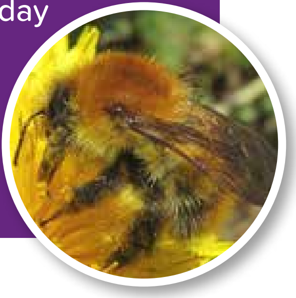

ON THE DAY

Ready to get started? It's time to begin:

- It's important to fill in all the data fields, so have information to hand that students might need (e.g. the school's postcode, locality etc.) You might want to take a look at the interactive data capture tool beforehand, so you know how it works.
- Hand out the tracking sheets or logbooks for students to fill in during the observation (if they're not directly uploading the information to the Pod's **Experiment Zone** via a laptop or mobile device).
- Make sure you have a stopwatch or timer to record the duration of the observation(s).
- It's unlikely to happen, but remember that bumblebees can sting, so warn students not to get too close or to touch the bees.
- **Upload your data to the Pod** (see ***over***): otherwise you won't be able to contribute to the research!
- Enjoyed the experiment? Make time to do more observations before the end of August.

THE Pod jointhepod.org

EDF ENERGY

Energy | Science | Sustainability

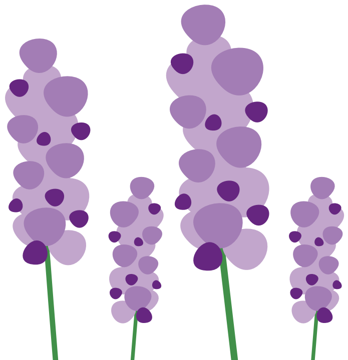

ACT

Record your observations

After the experiment is over, it's time for the really important bit: uploading your school's results!

UPLOAD YOUR DATA TO THE POD

When the experiment is over, visit the **Experiment Zone** to upload your findings into the interactive data capture tool. It only takes a couple of minutes and you could use this as an opportunity to reinforce students' learning. For instance, ask students why the lavender's proximity to other flowers might have an impact on the number of bumblebees spotted. Or, how might the weather affect their experiment's results?

Tell us about your experiences

Post a **blog** and share your experiences of The Big Bumblebee Discovery with other schools. It's a great way to tell other schools, students, and families what your school is doing. You can also **search the blogs** to get advice from other schools on running the experiment and discover their top tips.

Try another Pod activity

If your students are buzzing about biodiversity afterwards, why not explore some of the Pod's other resources? Share the facts in the **biodiversity information pack** or download the **bug hunt lesson plan**. If learning about the decline in bumblebees has inspired students to learn more about environmental change, take a look at the Pod's climate science resources, including the **carbon sources and sinks game** and **degrees of change lesson plan**.

Be a part of something big!

Build on what students have learnt by running the experiment again but, this time, adding a twist and competing against another class to see to see who can do the most observations. Visit the **map** on the Pod and zoom in to find which local schools near you are taking part. You can also see a heat map that shows which schools have entered data – are any near you? Why not try competing against one another? Perhaps you could link up on activities too; why not do an observation at a local secondary school, as they may have more outdoor space (if they haven't got any lavender, you could take along your pot)? Promote friendly rivalry with other Pod schools by challenging students to do as many observations as they can to boost their school's position on the leaderboard.

REWARD STUDENTS

- Hand out stickers to students when they upload their data– or when they correctly identify the colour groups.
- There are also certificates for students in the engagement pack – why not present these at an end of term assembly?
- When you've uploaded your data, you'll be able to 'unlock' an exciting new interactive reward game on the Pod. Why not hold a class or school competition to see who can complete it in the fastest time?

Look out for a **new video** in the summer following students taking part in the Big Bumblebee Discovery.

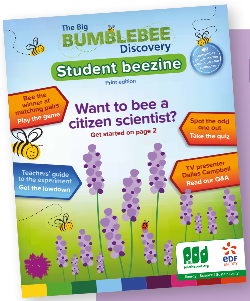

Don't forget about your lavender after the experiment! If you haven't planted it already, now is a good time to do so. We also have suggestions for how to use the lavender flowers afterwards in the **beezine**.

THE pod jointhepod.org

EDF ENERGY

Energy | Science | Sustainability

INSPIRE

Encourage families, friends and community groups to join in

Continue the experiment over the holidays and inspire others to take part

RUN THE EXPERIMENT IN THE HOLIDAYS

Hand out logbooks for students to carry out some observations in their garden – or in the park if they don't have any lavender in their own garden – and remind them to upload their results afterwards. Anyone can take part – parents, families, friends and carers – but every participant must log the essential details in the **Experiment Zone** for their observations to count towards this valuable research. To plan ahead, parents can download the **discovery pack** for useful hints and tips.

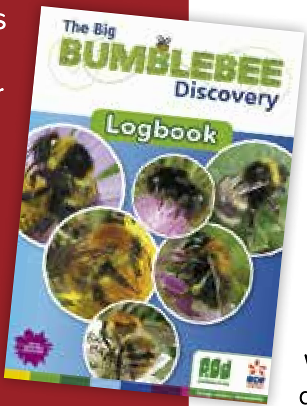

Battle of the bumblebees

Why not encourage students to re-do the experiment at home with their families and and compare their findings with a friend's household? This could work really well against a family that lives in another area of the UK with different environmental conditions. Students could do a presentation about what they spotted – and any differences in their results – after the summer break.

Be social

Teachers or parents could share students' observation and bumblebee-inspired work on social media sites, like Facebook and Twitter (**#beediscovery**). Why not set up a school Pinterest page to display students' prize spots? Parents can also use social media to organise their own events, where the wider community can get involved in citizen science too.

ARE YOU A MEMBER OF A HOLIDAY CLUB?

Why not encourage them to take part in The Big Bumblebee Discovery too? They can **sign up** and **download the resources** they need from the Pod. Don't forget to upload your results afterwards – it all counts towards the scientific research.

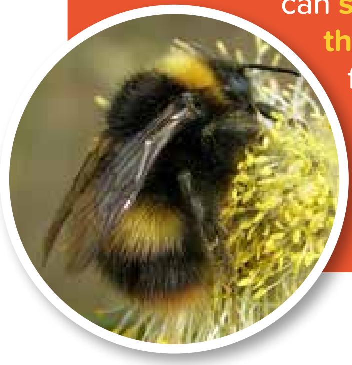

> I've always been a **'why' child** and if you're that kind of person, **science is a really great track** [for you]. You can go home at the end of the day and know you've contributed to something **bigger than yourself**.
>
> Jessica Batts
> EDF Energy young engineer

Don't forget to write your school's **blog code** on the inside of their logbooks (log in and find this on your School page). Students need this for linking their observations to your school and posting any blogs over the summer holidays.

THE Pod jointhepod.org

EDF ENERGY

Energy | Science | Sustainability

Inspire tomorrow's scientists

Maintain momentum in science and cultivate budding scientists with these further activity ideas

Get busy bio-blitzing

A 'bio-blitz' is an easy follow-up activity to The Big Bumblebee Discovery. All students need to do is see how many different types of wildlife ('species') they can find in a certain time period. Why not repeat this in different ecological environments ('habitats'), such as woods, by water or gardens? For an even simpler take on this activity, tap on a tree branch and see what bugs fall into an upturned umbrella positioned underneath – this is one of Dr Helen Roy's favourite activities! You can check the identifiction of any species you see by posting a photo on iSpot (www.ispotnature.org). Why not post your observations on iRecord (www.brc.ac.uk/irecord/) too? Both sites are dedicated to making it easier for people to record their own observations from nature.

Take part in a national survey

It's not just bumblebees that researchers are crying out for young scientists to track. They are also keen to receive data about biodiversity in general. If students want to take part in a bigger bio-blitz, which feeds data back into live research, they could take part – as individuals or as a class – in national surveys. Bio-blitz events are run all over the country; for instance, the BBC lists ongoing science projects requiring volunteers at: www.bbc.co.uk/nature/22694347.

In search of animal signs

You can find lots of clues in nature that indicate animals have been nearby. Why not embark on a nature walk, in search of animal signs, like footprints, holes, feathers, hair stuck on wire fences and, of course, poo! For tracking tips and ideas for what to spot at different times of the year, take a look at www.woodlandtrust.org.uk/learn/children-and-families/ and www.discoverwildlife.com/british-wildlife – and remind students of the importance of not touching animal faeces, and washing their hands properly after their nature walk.

INSPIRING STUDENT SCIENTISTS

In 2010, pupils from Blackawton School in Devon made history when a scientific paper they'd written on an experiment involving bumblebees was published in a highly respected academic journal. Their discovery? That bumblebees use the colour and pattern of flowers when seeking out pollen. Find out more at www.lottolab.org/articles/blackawtonbees.asp

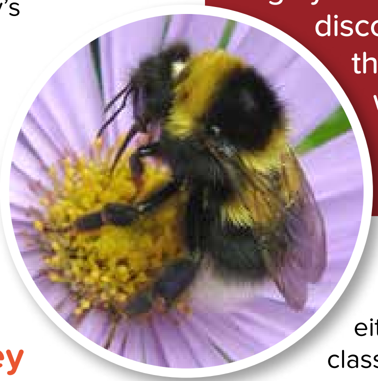

This activity can be done by students either as individuals, families or as a class. The activity could also be linked into literacy objectives by getting students to describe smell, appearance, colour or write a story about the animal(s) they've tracked.

Superhero scientists

Why not reward students' interest in citizen science with a simple ranking system? For instance, after taking part in The Big Bumblebee Discovery each student could be promoted from level 1 ('Newbie Scientist') to level 2 ('The Lab Apprentice'). Depending how many tasks and extra activities they take part in, students could move up the science career ladder to achieve the top ranking: 'Practically Albert(a) Einstein'! Keeping track of these rankings could be as simple as moving names up a drawing of a ladder on your classroom wall. Or you could tie the task into literacy objectives by getting each student to design their own scientist icon, which could move up the ladder. The more tech-savvy may even want to design their personal avatar on a computer, adding extra accessories, as they progress through the ranks.

Useful resources

Quick links to all The Big Bumblebee Discovery resources you need

Pod resources:

Sign up to the experiment:
www.jointhepod.org/experiment-zone

Download experiment resources
www.jointhepod.org/experimentresources

Upload your experiment results
www.jointhepod.org/experiment-zone

Blog about the experiment
www.jointhepod.org/beeblog

Download take home resources
www.jointhepod.org/beetakehomesheets

Find other schools taking part
www.jointhepod.org/pod-community/map

Download other biodiversity resources
www.jointhepod.org/biodiversityresources

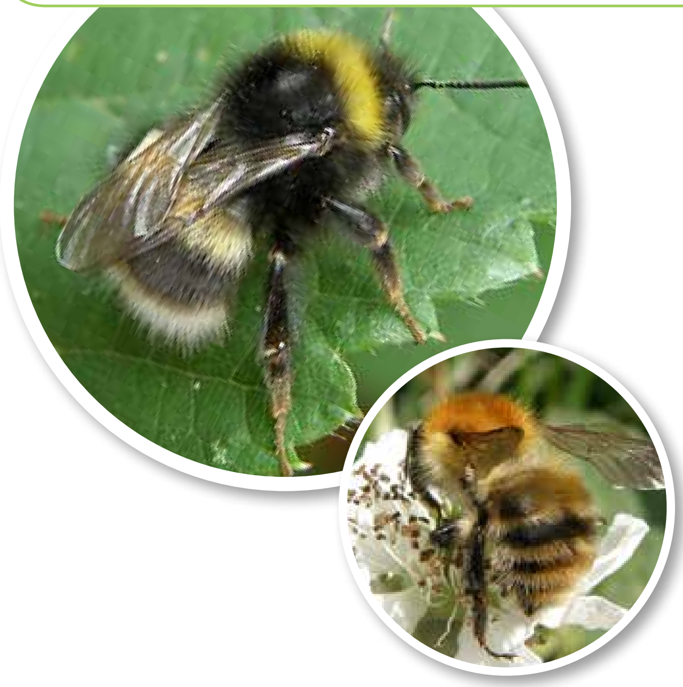

What happens next?

Scientists at the Centre For Ecology & Hydrology will analyse your data to see what it tells us about bumblebee populations. We will share these results with you on the Pod. Our longer term aim is to publish a scientific paper about the study. This can take several months, so is likely to happen in 2015.
