# Supplementary material for: Focal Plant Observations as a Standardised Method for Pollinator Monitoring: Opportunities and Limitations for Mass Participation Citizen Science
Source: PLoS One. 2016 Mar 17;11(3):e0150794. doi: 10.1371/journal.pone.0150794 (PMC4795797; doi:10.1371/journal.pone.0150794)
Supplement: S2 Table — (DOCX) [file pone.0150794.s006.docx]

## S3 Details of the classification matrices of colour types of bumblebees.

Number of classifications (% of actual identification across submitted identifications in brackets) for photographs submitted as part of the Big Bumblebee Discovery. Precision is the percentage of records submitted as such that were identified correctly. Miss rate is the percentage of records of that type which were incorrectly identified (miss rate% = 100 – sensitivity%).

|  |  | Actual identification | | | | | | | | | | | |  | |  |  |  |  |
| --- | --- | --- | --- | --- | --- | --- | --- | --- | --- | --- | --- | --- | --- | --- | --- | --- | --- | --- | --- |
|  |  | Banded orange tail | Black-bodied red tail (female)* | Black-bodied red tail (male)* | Brown | Orange white-tail | Two-banded white tail | Three-banded white tail | *Bombus vestalis***** | Unknown | Hoverfly species (Syrpidae) | Honeybee (*Apis* *mellifera*) | No bee present¶ | | Total | | Precision (of the colour type reported) | | Precision (of being a bumblebee) |
| Submitted identification | Banded orange tail | 5 (31) | 1 (6) | 1 (6) |  |  | 1 (6) |  |  | 3 (19) | 2 (13) | 1 (6) | 2 | | 14 | | 36 | | 50 |
|  | Black-bodied orange tail |  | 6 (86) |  |  |  |  |  |  | 1 (14) |  |  |  | | 7 | | 86 | | 86 |
|  | Brown | 1 (20) |  | 1 (20) |  |  | 1 (20) |  |  | 2 (40) |  |  |  | | 5 | | 0 | | 60 |
|  | Orange white-tail |  |  |  | 15 (63) | 2 (8) | 1 (4) |  |  |  | 3 (13) | 3 (13) |  | | 24 | | 8 | | 75 |
|  | Two-banded white tail |  |  |  |  |  | 21 (81) | 2 (4) | 1 (4) |  |  |  | 2 | | 24 | | 88 | | 92 |
|  | Three-banded white tail |  |  |  |  |  |  |  |  |  |  |  |  | | 0 | | NA | | NA |
|  | Unknown |  |  | 1 (20) |  | 2 (40) |  |  |  | 1 (20) | 1 (20) |  |  | | 5 | |  | |  |
|  | Total | 6 | 7 | 3 | 15 | 4 | 24 | 2 | 1 | 7 | 6 | 4 |  | | 83 | |  | |  |
|  | Miss rate (in %) | 17 | 14 | 100 | 100 | 50 | 12 | 0 |  |  |  |  |  | |  | |  | |  |

* Female and male *Bombus lapidarius* (black-bodied orange tail) are separated out because they are very distinctive, but are combined for the calculation of accuracy. Overall miss rate for females and males combined was 40%.

** *Bombus vestalis* is separated out because it was the commonest species which did not fit neatly into the six colour type categories (Table 1).

¶ Photographs with no bee present were excluded from the totals and the calculation of accuracy.

Number of classifications (% of actual identification across submitted identifications in brackets) for photographs identified during the identification at the British Science Festival 2015. Precision is the percentage of records submitted as such that were identified correctly. Miss rate is the percentage of records of that type which were incorrectly identified (miss rate% = 100 – sensitivity%).

|  |  | Actual identification | | | | | | | | | | | |  | |  |  |  |  |
| --- | --- | --- | --- | --- | --- | --- | --- | --- | --- | --- | --- | --- | --- | --- | --- | --- | --- | --- | --- |
|  |  | Banded orange tail | Black-bodied red tail (female)* | Black-bodied red tail (male)* | Brown | Orange white-tail | Two-banded white tail | Three-banded white tail | *Bombus vestalis*** | Honeybee (*Apis* *mellifera*) | Hoverfly (Syrpidae: *Eristalis* sp) | Social wasp | Butterfly | | Total | | Precision (of the colour type reported) | | Precision (of being a bumblebee) |
| Submitted identification | Banded orange tail | 86 (61) | 2 (1) | 16 (11) | 2 (1) | 1 (1) | 18 (13) | 11 (8) |  | 3 (2) | 2 (1) |  |  | | 142 | | 61 | | 96 |
|  | Black-bodied orange tail | 8 (11) | 49 (70) | 7 (10) |  |  | 3 (4) |  |  | 1 (1) | 2 (3) |  |  | | 70 | | 80 | | 96 |
|  | Brown | 4 (5) |  |  | 18 (23) | 1 (1) | 1 (1) |  |  | 23 (29) | 33 (41) |  |  | | 80 | | 23 | | 30 |
|  | Orange white-tail | 4 (7) |  |  | 1 (2) | 30 (56) | 2 (4) | 2 (4) | 6 (11) | 1 (2) | 4 (7) | 4 (7) |  | | 54 | | 56 | | 83 |
|  | Two-banded white tail | 13 (10) |  | 1 (1) | 1 (1) | 5 (4) | 92 (72) | 15 (12) |  | 1 (1) |  |  |  | | 128 | | 72 | | 99 |
|  | Three-banded white tail | 6 (5) |  |  | 1 (1) | 5 (4) | 28 (22) | 67 (53) | 19 (15) | 1 (1) |  |  |  | | 127 | | 53 | | 99 |
|  | Unknown bumblebee | 9 (23) |  | 1 (3) | 2 (5) | 1 (3) | 3 (8) | 2 (5) | 2 (5) | 5 (13) | 11 (28) | 3 (8) | 1 (3) | | 40 | |  | |  |
|  | Unknown | 5 (3) | 1 (1) | 2 (1) | 2 (1) | 6 (4) | 14 (9) | 8 (5) |  | 19 (12) | 57 (36) | 20 (13) | 25 (16) | | 159 | |  | |  |
|  | Total | 135 | 52 | 27 | 27 | 49 | 161 | 105 | 27 | 54 | 109 | 27 | 27 | | 83 | |  | |  |
|  | miss rate (in %) | 36 | 6 | 74 | 33 | 39 | 36 | 43 |  |  |  |  |  | |  | |  | |  |

* Female and male *Bombus lapidarius* (black-bodied orange tail) are separated out because they are very distinctive, but are combined for the calculation of accuracy. Overall miss rate for females and males combined was 29%.

* *Bombus vestalis* is treated separately because it was the commonest species which did not fit neatly into the six colour type categories (Table 1).
